# Supplementary material for: Assessing the impact of group antenatal care on gestational length in Rwanda: A cluster-randomized trial
Source: PLoS One. 2021 Feb 2;16(2):e0246442. doi: 10.1371/journal.pone.0246442 (PMC7853466; doi:10.1371/journal.pone.0246442)
Supplement: S1 Table — (DOCX) [file pone.0246442.s001.docx]

**S1 Table. Gestational length, incidence of preterm birth, and incidence of low birth weight among a subset of women; gestational length calculated by ultrasound-adjusted gestational age when ultrasound examination was completed between 6 and 22 weeks gestation.**

|  | **Control** | | **Intervention** | | **P-value*** |
| --- | --- | --- | --- | --- | --- |
|  | **n** | **Mean (SE) / %** | **n** | **Mean (SE) / %** |  |
| Gestational length (in weeks) | 765 | 39.5 (0.03) | 614 | 39.8 (0.03) | 0.78 |
| Preterm birth | 765 | 8.6 | 614 | 7.5 | 0.20 |
| Low birth weight | 747 | 4.0 | 609 | 4.4 | 0.63 |

*****P-values were calculated using generalized estimating equations (GEE) with robust variance estimation to account for clustering of births within facility and to adjust for pairing of facilities.

n_=_Total number of non-missing observations for the respective variable in the control/intervention arm
