# Supplementary material for: Assessing the impact of group antenatal care on gestational length in Rwanda: A cluster-randomized trial
Source: PLoS One. 2021 Feb 2;16(2):e0246442. doi: 10.1371/journal.pone.0246442 (PMC7853466; doi:10.1371/journal.pone.0246442)
Supplement: S2 Table — (DOCX) [file pone.0246442.s002.docx]

**S2 Table. Adjusted analysis for maternal characteristics associated with selected outcomes, using the control group as the reference**

| Woman's characteristic | Outcome | Beta / OR | p-value | Lower limit | Upper limit |
| --- | --- | --- | --- | --- | --- |
| Pregnant woman smokes tobacco | GA | -0.06 | 0.27 | -0.18 | 0.05 |
|  | PTB | 1.04 | 0.84 | 0.72 | 1.51 |
|  | Cesarean birth | 1.00 | 0.99 | 0.78 | 1.28 |
|  | Multiple gestation | 1.04 | 0.85 | 0.67 | 1.62 |
|  | LBW | 1.28 | 0.10 | 0.95 | 1.71 |
| Someone in the pregnant woman’s household smokes tobacco | GA | -0.07 | 0.24 | -0.18 | 0.05 |
|  | PTB | 1.05 | 0.79 | 0.72 | 1.53 |
|  | Cesarean birth | 1.00 | 1.00 | 0.78 | 1.27 |
|  | Multiple gestation | 1.05 | 0.82 | 0.68 | 1.64 |
|  | LBW | 1.28 | 0.11 | 0.95 | 1.72 |
| Pregnant woman drinks alcohol | GA | -0.07 | 0.26 | -0.18 | 0.05 |
|  | PTB | 1.03 | 0.89 | 0.71 | 1.50 |
|  | Cesarean birth | 1.00 | 0.97 | 0.78 | 1.27 |
|  | Multiple gestation | 1.04 | 0.86 | 0.67 | 1.61 |
|  | LBW | 1.27 | 0.11 | 0.94 | 1.70 |
| Pregnant woman's age at the first ANC visit | GA | -0.07 | 0.22 | -0.18 | 0.04 |
|  | PTB | 1.05 | 0.81 | 0.73 | 1.51 |
|  | Cesarean birth | 1.01 | 0.91 | 0.79 | 1.30 |
|  | Multiple gestation | 1.07 | 0.76 | 0.69 | 1.66 |
|  | LBW | 1.30 | 0.08 | 0.97 | 1.74 |
| Pregnant woman uses solid fuel to cook indoors | GA | -0.07 | 0.24 | -0.18 | 0.05 |
|  | PTB | 1.06 | 0.77 | 0.72 | 1.55 |
|  | Cesarean birth | 0.99 | 0.95 | 0.78 | 1.26 |
|  | Multiple gestation | 0.90 | 0.59 | 0.62 | 1.32 |
|  | LBW | 1.26 | 0.15 | 0.92 | 1.73 |
| Pregnant woman weighed less than 45 or more than 80 kilograms at first ANC visit | GA | -0.09 | 0.12 | -0.20 | 0.02 |
|  | PTB | 1.10 | 0.62 | 0.76 | 1.59 |
|  | Cesarean birth | 1.01 | 0.92 | 0.79 | 1.30 |
|  | Multiple gestation | 1.10 | 0.71 | 0.67 | 1.81 |
|  | LBW | **1**.**34** | **0.049*** | **1.002** | **1.79** |
| Pregnant woman's height less than 150 cm | GA | -0.08 | 0.16 | -0.19 | 0.03 |
|  | PTB | 1.08 | 0.68 | 0.74 | 1.59 |
|  | Cesarean birth | 1.01 | 0.91 | 0.79 | 1.30 |
|  | Multiple gestation | 1.04 | 0.86 | 0.67 | 1.63 |
|  | LBW | 1.33 | 0.07 | 0.98 | 1.80 |
| Pregnant woman's middle-upper arm circumference less than 21 cm at first ANC visit | GA | -0.07 | 0.25 | -0.18 | 0.05 |
|  | PTB | 1.04 | 0.82 | 0.72 | 1.51 |
|  | Cesarean birth | 1.00 | 0.98 | 0.78 | 1.27 |
|  | Multiple gestation | 1.05 | 0.82 | 0.68 | 1.63 |
|  | LBW | 1.31 | 0.07 | 0.98 | 1.76 |
| Pregnant woman reported a past pregnancy history of PTB, LBW, stillbirth, or neonatal death | GA | -0.07 | 0.23 | -0.18 | 0.04 |
|  | PTB | 1.07 | 0.72 | 0.74 | 1.55 |
|  | Cesarean birth | 1.00 | 0.99 | 0.78 | 1.28 |
|  | Multiple gestation | 1.05 | 0.85 | 0.67 | 1.62 |
|  | LBW | 1.32 | 0.06 | 0.99 | 1.78 |
| Pregnant woman in the 2 lowest Ubudehe categories** | GA | -0.06 | 0.32 | -0.17 | 0.05 |
|  | PTB | 1.18 | 0.47 | 0.76 | 1.81 |
|  | Cesarean birth | 1.02 | 0.88 | 0.78 | 1.34 |
|  | Multiple gestation | 0.84 | 0.54 | 0.48 | 1.47 |
|  | LBW | 1.18 | 0.31 | 0.86 | 1.62 |
| Pregnant woman did not complete primary education | GA | -0.08 | 0.17 | -0.19 | 0.03 |
|  | PTB | 1.06 | 0.75 | 0.74 | 1.53 |
|  | Cesarean birth | 0.97 | 0.77 | 0.77 | 1.21 |
|  | Multiple gestation | 1.03 | 0.88 | 0.67 | 1.60 |
|  | LBW | 1.29 | 0.10 | 0.95 | 1.75 |
| Pregnant woman gave birth to multiples in this pregnancy | GA | -0.06 | 0.31 | -0.17 | 0.05 |
|  | PTB | 1.05 | 0.81 | 0.72 | 1.52 |
|  | Cesarean birth | 1.04 | 0.74 | 0.82 | 1.32 |
|  | Multiple gestation | NA | ·· | ·· | ·· |
|  | LBW | **1**.**55** | **0.002*** | **1.17** | **2.06** |
| Woman's HIV status was known positive before testing at first ANC visit | GA | -0.024 | 0.70 | -0.145 | 0.097 |
|  | PTB | 0.947 | 0.77 | 0.655 | 1.368 |
|  | Cesarean birth | 1.019 | 0.90 | 0.774 | 1.341 |
|  | Multiple gestation | 0.903 | 0.66 | 0.577 | 1.415 |
|  | LBW | 1.37 | 0.06 | 0.99 | 1.88 |
| Woman's HIV test result at first ANC visit was positive | GA | -0.06 | 0.33 | -0.18 | 0.06 |
|  | PTB | 0.93 | 0.73 | 0.61 | 1.41 |
|  | Cesarean birth | 0.98 | 0.86 | 0.77 | 1.25 |
|  | Multiple gestation | 1.04 | 0.86 | 0.66 | 1.65 |
|  | LBW | 1.30 | 0.06 | 0.99 | 1.72 |
| Woman stated she was nulliparous at first ANC visit | GA | -0.07 | 0.22 | -0.19 | 0.04 |
|  | PTB | 1.05 | 0.79 | 0.73 | 1.52 |
|  | Cesarean birth | 0.97 | 0.77 | 0.77 | 1.22 |
|  | Multiple gestation | 1.11 | 0.64 | 0.71 | 1.75 |
|  | LBW | 1.14 | 0.51 | 0.76 | 1.71 |

*Two-tailed test, α=0.05.

**An Ubedehe category is assigned to each household. Local community members at the cell level are required to gather community members together and, with the help of Ubudehe facilitators/trainers, identify and place community members into different economic categories, ranging from the poorest households (Category 1) to the richest households (Category 4).^21^

Acronyms: Gestational age (GA), antenatal visit (ANC), preterm birth (PTB), low birth weight (LBW), human immunodeficiency virus (HIV)
