# Supplementary material for: Assessing the impact of group antenatal care on gestational length in Rwanda: A cluster-randomized trial
Source: PLoS One. 2021 Feb 2;16(2):e0246442. doi: 10.1371/journal.pone.0246442 (PMC7853466; doi:10.1371/journal.pone.0246442)
Supplement: S3 Table — (DOCX) [file pone.0246442.s003.docx]

**S3 Table. Distribution of secondary outcomes by study groups and the effect of the intervention on these outcomes.**

|  | **Control** | | **Intervention** | | **p-value*** |
| --- | --- | --- | --- | --- | --- |
|  | **n_1/_n_2_** | **% / Mean (SE)** | **n_1/_n_2_** | **% / Mean (SE)** |  |
| Low birth weight | 88/4129 | 2.1 | 119/4784 | 2.5 | 0.07 |
| Proportion of women who attended at least 3  ANC visits | 5434/7579 | 71.7 | 6665/8259 | 80.7 | 0.003 |
| Mean number of ANC visits | 7579 | 3.05 (0.06) | 8259 | 3.19 (0.04) | 0.05 |
| Mean gestational age at first ANC visit, in  weeks | 7516 | 15.8 (0.31) | 8223 | 16.0 (0.26) | 0.67 |
| Proportion of these women who attended  ANC 1 before 16 completed weeks gestation | 4525/7516 | 60.2 | 4457/8223 | 54.2 | 0.27 |

*****P-values were calculated using generalized estimating equations (GEE) with robust variance estimation to account for clustering of births within facility and to adjust for pairing of facilities.

n_1_=Numerator for the specific category in the control/intervention arm,

n_2_=Total number of non-missing observations for the respective variable in the control/intervention arm
