# Supplementary material for: Assessing the impact of group antenatal care on gestational length in Rwanda: A cluster-randomized trial
Source: PLoS One. 2021 Feb 2;16(2):e0246442. doi: 10.1371/journal.pone.0246442 (PMC7853466; doi:10.1371/journal.pone.0246442)
Supplement: S2 File — (DOCX) [file pone.0246442.s005.docx]

#### Study Application (Version 1.18)

| ***Enter the full title of your study:** | | |
| --- | --- | --- |
| UCSF Preterm Birth Initiative East Africa: Group Antenatal/Postnatal Care in Rwanda |  |  |
| ***Enter the study number or study alias** | | |
| PTBi East Africa Rwanda  * This field allows you to enter an abbreviated version of the Study Title to quickly identify this study. |  |  |

| **Primary Dept?** | **Department Name** |
| --- | --- |
| ◆ | **UCSF** - *482218* - H_GHS_PROGRAM_PN_MH |
|  | **UCSF** - *482201* - H_GHS - Central Admin |

| **1.0 General Information** | | |
| --- | --- | --- |
|  | | |
| **2.0 Add Department(s)** | | |
| **2.1 List the departments associated with this study. The Principal Investigator's department should be Primary.:** | |  |
| 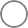 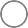 |  |  |
| **3.0 List the key study personnel: (Note: external and affiliated collaborators who are not in the UCSF directory can be identified later in the Qualifications of Key Study Personnel section at the end of the form)** | | |
| **3.1 *Please add a Principal Investigator for the study:** | |  |
| Walker, Dilys, MD  Select if applicable  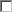 Department Chair 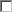 Resident  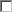 Fellow  If the Principal Investigator is a Fellow, the name of the Faculty Advisor must be supplied below. |  |  |
| **3.2 If applicable, please select the Research Staff personnel:** | |  |
| A) Additional Investigators |  |  |
|  |  |  |
| B) Research Support Staff |  |  |
| Azman Firdaus, Hana M Data Manager  Butrick, Elizabeth A Study Coordinator |  |  |

| Ghosh, Rakesh Biostatistician Lundeen, Tiffany B Study Coordinator Millar, Kathryn R  Volunteer/Student Intern (through UCSF Medical Center) Miller, Phoebe N  Volunteer/Student Intern (through UCSF Medical Center) Myrick, Roger  Data Manager Phillips, Elizabeth S Data Manager Santos, Nicole M Study Coordinator Schmidt, Christina N Research Assistant Sloan, Nancy L Biostatistician Sparks, Aleah Research Assistant Sterling, Mona A UCSF Core Personnel Williams, Pamela G  Volunteer/Student Intern (through UCSF Medical Center) |  |  |
| --- | --- | --- |
| **3.3 *Please add a Study Contact:** | |  |
| Butrick, Elizabeth A Lundeen, Tiffany B Phillips, Elizabeth S Santos, Nicole M Sterling, Mona A Walker, Dilys, MD  The Study Contact(s) will receive all important system notifications along with the Principal Investigator. (e.g. The project contact(s) are typically either the Study Coordinator or the Principal Investigator themselves). |  |  |
| **3.4 If applicable, please add a Faculty Advisor/Mentor:** | |  |
|  |  |  |
| **3.5 If applicable, please select the Designated Department Approval(s):** | |  |
| Add the name of the individual authorized to approve and sign off on this protocol from your Department (e.g. the Department Chair or Dean). |  |  |
| **4.0**  **Initial Screening Questions**  **Updated January 2019 - Revised Common Rule (January 2018) Compliant - v92** | | |
| **4.1 * PROJECT SUMMARY: (REQUIRED) Give a brief overview of this project (250 words or less). Tell us** | | |

| **what this study is about, who is being studied, and what it aims to achieve. If you have an NIH Abstract, paste it here (Click on the orange question mark to the right for more detailed instructions):** | |
| --- | --- |
| The Preterm Birth Initiative East Africa (PTBi EA) is a partnership between the Bill & Melinda Gates Foundation (BMGF) and the University of California, San Francisco (UCSF). In Rwanda, PTBi EA is working in collaboration with the University of Rwanda and the Ministry of Health (MOH). The focus of this work is to improve antenatal care (ANC) and postnatal care (PNC) at the health center level in five districts (Bugesera, Burera, Nyamasheke, Nyarugenge, and Rubavu), in which we have selected 36 health centers. This application reflects the second phase of our efforts in which we plan to conduct a cluster randomized control trial (RCT) of group ANC and PNC care to measure its effectiveness on increasing gestational age, mortality among preterm and low birth weight infants, as well as adherence to focused ANC and PNC practices. We hypothesize that group ANC and PNC implemented at the health center level will increase gestational age at birth among women who receive group care compared to women who receive standard focused ANC. To improve assessment of gestational age, we will also introduce ultrasound at the health center level for half of our facilities. These facilities will also incorporate pregnancy testing with urine dipstick to be performed by community health workers in charge of maternal health to facilitate early entry into ANC. If this work shows positive outcomes, we hope to support scale-up activities. |  |
| **4.2 * HUD DEVICE: (REQUIRED) Does this application involve a** [**Humanitarian Use Device**](https://www.fda.gov/medicaldevices/deviceregulationandguidance/howtomarketyourdevice/premarketsubmissions/humanitariandeviceexemption/default.htm) **(HUD):** | |
| 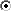 No  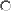 Yes, and it includes a research component 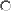 Yes, and it involves clinical care ONLY |  |
| **4.3 * TYPE OF RESEARCH: (REQUIRED) Select the option that best fits your project (Click the orange question mark to the right for definitions and guidance):** | |
| 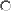 Biomedical research (including medical records review, biospecimen collection and/or use, other healthcare or health outcomes related activities, research database, biospecimen bank, or recruitment registry)  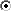 Social, behavioral, educational, and/or public policy research  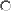 Hybrid - includes aspects of BOTH types of research (check this option if your research is mainly social  /behavioral but also involves specimen collection or blood draws to look at biological measures) |  |
| **4.4 * SUBJECT CONTACT: (REQUIRED) Does this study involve ANY contact or interactions with participants:** | |
| 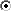 Yes (including phone, email or web contact)  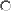 No (limited to medical records review, biological specimen analysis, and/or data analysis) |  |
| **4.5 * RISK LEVEL: (REQUIRED) What is your estimation of the risk level, including all screening procedures and study activities:** | |
| 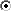 Minimal risk  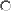 Greater than minimal risk |  |
| **4.6 * REVIEW LEVEL: (REQUIRED) Requested review level (Click on the orange question mark to the right for definitions and guidance):** | |
| 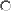 Full Committee 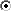 Expedited  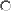 Exempt |  |
| **4.7 * EXPEDITED REVIEW CATEGORIES: (REQUIRED) If you think this study qualifies for expedited** | |

| **review, select the** [**regulatory categories**](http://irb.ucsf.edu/levels-review#expedited) **that the research falls under: (check all that apply)** | |
| --- | --- |
| 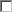 Category 1: A very limited number of studies of approved drugs and devices 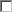 Category 2: Blood sampling  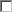 Category 3: Noninvasive specimen collection (e.g. buccal swabs, urine, hair and nail clippings, etc.)  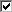 Category 4: Noninvasive clinical procedures (e.g. physical sensors such as pulse oximeters, MRI, EKG, EEG, ultrasound, moderate exercise testing, etc.)  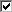 Category 5: Research involving materials (data, documents, records, or specimens) that were previously collected for either nonresearch or research purposes  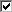 Category 6: Use of recordings (voice, video, digital or image)  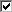 Category 7: Low risk behavioral research or research employing survey, interview, oral history, focus group, program evaluation, human factors evaluation, or quality assurance methodologies |  |
| **4.9 * DATA/SPECIMEN ANALYSIS ONLY: (REQUIRED) Does this study ONLY involve records review and**  **/or biospecimen analysis (do not check 'Yes' if this is a registry, research or recruitment database, or biospecimen repository):** | |
| 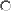 Yes 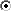 No |  |
| **4.10 * CLINICAL TRIAL: (REQUIRED) Is this a clinical trial:** | |
| **According to The World Health Organization (WHO) and the International Committee of Medical Journal Editors (ICMJE) a** [**clinical trial**](http://www.who.int/topics/clinical_trials/en/) **is:**  Any research study that prospectively assigns human participants or groups of humans to one or more health-related interventions to evaluate the effects on health outcomes.  ICMJE requires registration of a clinical trial in a public database (such as ClinicalTrials.gov) prior to enrollment, for eventual publication of results in member biomedical journals.  **Guidance:** Public Law 110-85 requires that all investigators who perform an *applicable clinical trial* must ensure that the trial is registered on a government web site called [**ClinicalTrials.gov**](http://www.clinicaltrials.gov/).  **The FDA requires registration for 'applicable clinical trials,' defined as follows:**  For any trials of drugs and biologics: controlled clinical investigations, other than Phase 1 investigations, of a product subject to FDA regulation.  For trials of biomedical devices: controlled trials with health outcomes of devices subject to FDA regulation, other than small feasibility studies, and pediatric post-market surveillance.  For additional information on the [**ClinicalTrials.gov**](http://www.clinicaltrials.gov/) registration process at UCSF and the definition of a clinical trial for purposes of registration, visit the [**ClinicalTrials.gov section of the UCSF Clinical Research Resource HUB**](http://hub.ucsf.edu/clinicaltrialsgov%20%20).  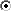 Yes 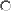 No  **Clinical Trial Registration** - 'NCT' number for this trial:  NCT03154177 |  |

| **4.11 * CLINICAL TRIAL PHASE: (REQUIRED) Check the applicable phase(s):** | |
| --- | --- |
| 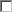 Phase 0  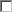 Phase 1 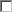 Phase 1/2 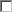 Phase 2 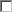 Phase 2/3 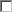 Phase 3  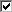 Phase 4  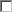 Not Applicable |  |
| **4.12 * INVESTIGATOR-INITIATED: (REQUIRED) Is this an investigator-initiated study:** | |
| 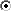 Yes 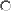 No  **The UCSF IRB recommends use of the** [**Virtual Regulatory Binder**](https://hub.ucsf.edu/virtual-regulatory-binder) **to manage your study.** |  |
| **4.13 * CANCER: (REQUIRED) Does this study involve cancer (e.g., the study involves patients with cancer or at risk for cancer, including behavioral research, epidemiological research, public policy research, specimen analysis, and chart reviews):** | |
| 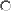 Yes 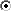 No |  |
| **4.14 * RADIATION EXPOSURE: (REQUIRED) Does your protocol involve any radiation exposure to patients**  **/subjects EITHER from standard care OR for research purposes (e.g., x-rays, CT-scans, DEXA, CT- guided biopsy, radiation therapy, or nuclear medicine including PET, MUGA or bone scans):** | |
| 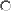 Yes 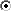 No |  |
| **4.15 SCIENTIFIC REVIEW: If this study has undergone scientific or scholarly review, please indicate which entity performed the review (check all that apply):** | |
| 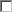 Cancer Center Protocol Review Committee (PRC) (Full approval is required prior to final IRB approval for cancer-related protocols.)  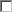 CTSI Clinical Research Services (CRS) Advisory Committee 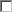 CTSI Consultation Services  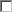 Departmental scientific review 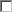 Other: |  |
| **4.16 * STEM CELLS: (REQUIRED) Does this study involve** [**human stem cells**](http://stemcells.nih.gov/info/basics/pages/basics1.aspx) **(including iPS cells and adult stem cells), gametes or embryos:** | |
| 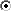 No  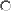 Yes, and requires IRB and GESCR review  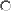 Yes, and requires GESCR review, but NOT IRB review |  |
| **4.17 * FINANCIAL INTERESTS: (REQUIRED) Do you or any other responsible personnel (or the spouse, registered domestic partner and/or dependent children thereof) have** [**financial interests**](http://coi.ucsf.edu/) **related to this study:** | |
| 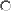 Yes 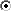 No |  |

| **View Details** | **Sponsor Name** | **Sponsor Type** | | **Awardee Institution** | **Contract Type:** | **UCSF RAS "P**  **number" or eProposal number** | **UCSF RAS**  **System Award Number ("A" + 6**  **digits)** |
| --- | --- | --- | --- | --- | --- | --- | --- |
|  | Bill & Melinda Gates Foundation | 06 | | UCSF | Grant |  | A123218 |
| Sponsor Name: | | | Bill & Melinda Gates Foundation | | | | |
| Sponsor Type: | | | 06 | | | | |
| Sponsor Role: | | | Funding | | | | |
| **Grant/Contract Number:** | | |  | | | | |
| Awardee Institution: | | | UCSF | | | | |
| **Is Institution the Primary Grant Holder:** | | | Yes | | | | |
| Contract Type: | | | Grant | | | | |
| UCSF RAS "P number" or eProposal number: | | |  | | | | |
| UCSF RAS System Award Number ("A" + 6 digits): | | | A123218 | | | | |
| Grant Number for Studies Not Funded thru UCSF: | | |  | | | | |
| Grant Title: | | | UCSF Preterm Birth Initiative | | | | |
| PI Name:  (If PI is not the same as identified on the study.) | | |  | | | | |
| Significant Discrepancy: | | |  | | | | |

| **5.0 Funding** | |
| --- | --- |
| **5.1 * FEDERAL FUNDING: (REQUIRED) Is this study currently supported in whole or in part by Federal funding, *even by a subcontract*, OR has it received ANY Federal funding in the past:** | |
| 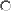 Yes 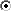 No |  |
| **5.2 * DoD INVOLVEMENT: Is this project linked in any way to the Department of Defense (DoD): (REQUIRED)** | |
| 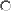 Yes 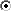 No |  |
| **5.3 SPONSORS: Identify all sponsors and provide the funding details. If funding comes from a Subcontract, please list only the Prime Sponsor:** | |
| **External Sponsors:**  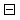  **Other Funding Sources and Unfunded Research - Gift, Program, Departmental or other Internal Funding (check all that apply):**  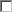 Funded by gift (specify source below)  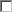 Funded by UCSF or UC-wide program (specify source below) 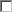 Specific departmental funding (specify source below) |  |

| 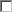 Unfunded (miscellaneous departmental funding) 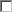 Unfunded student project |  |
| --- | --- |
| **6.0 Sites, Programs, Resources, and External IRB Review** | |
| **6.1 UCSF AND AFFILIATED SITES (check all that apply):** | |
| 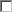 UCSF Benioff Children's Hospital Oakland (BCHO) 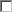 UCSF China Basin clinics and facilities  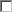 UCSF Helen Diller Family Comprehensive Cancer Center 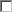 UCSF Langley Porter Psychiatric Institute (LPPI)  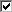 UCSF Medical Center at Mission Bay (Benioff Children's Hospital, the Betty Irene Moore Women's Hospital, Bakar Cancer Hospital, or outpatient clinics)  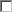 UCSF Mount Zion  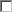 UCSF Parnassus (Moffitt-Long hospital, dental clinics or other outpatient clinics)  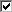 UCSF Other Sites (including Laurel Heights and all the other sites outside the main hospitals) 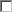 Zuckerberg San Francisco General (ZSFG)  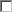 SF VA Medical Center (SF VAMC)  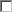 Fresno - UCSF Fresno OR Community Medical Center (CMC) 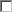 Gladstone  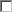 Institute on Aging (IOA) 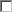 Jewish Home  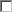 SF Dept of Public Health (DPH)  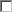 Vitalant (formerly Blood Centers of the Pacific and Blood Systems Research Institute) |  |
| **6.2 LOCATIONS: At what locations will study visits and activities occur:** | |
| The randomized control trial will take place across 36 health facilities and 6 district hospitals in the Burera, Bugesera, Nyamasheke, Rubavu, and Nyarugenge districts of Rwanda. Ultrasound will be introduced at half of the selected health centers with early pregnancy testing within the corresponding catchment areas. |  |
| **6.3 OFF-SITE PROCEDURES: Will any study procedures or tests be conducted off-site by non-UCSF personnel:** | |
| 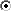 Yes 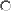 No  Please identify which procedures may be done off-site:  All study activities and data collection will take place in Rwanda. Data collection and implementation activities will be conducted by PTBi Rwanda staff hired University of Rwanda and Rwanda Biomedical Center (UoR and RBC, subcontractors). UCSF will provide technical assistance around data strengthening, group care training, and data analysis. The overall work will be a collaborative  among UCSF, UoR and RBC. |  |
| **6.4 RESEARCH PROGRAMS: Check any UCSF research programs this study is associated with:** | |
| 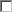 Cancer Center  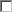 Center for AIDS Prevention Sciences (CAPS) 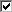 Global Health Sciences  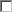 Immune Tolerance Network (ITN)  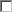 Neurosciences Clinical Research Unit (NCRU) 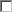 Osher Center  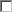 Positive Health Program |  |

|  |  |
| --- | --- |
| **6.5 * CTSI CRS SERVICES: (REQUIRED) Will this study be carried out at one of the** [**UCSF Clinical Research Services (CRS)**](https://accelerate.ucsf.edu/research/crs) **units or utilize** [**CRS services**](https://accelerate.ucsf.edu/research/crs)**:** | |
| 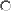 Yes 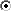 No |  |
| **6.6 * MULTI-CENTER TRIAL: (REQUIRED) Is this a multi-center or multi-site research trial:** | |
| By **'multi-center trial**' we mean a study where the protocol is developed by an lead investigator, an industry sponsor, consortium, a disease-group, etc.,and multiple sites across the nation or in different countries participate in the trial. The local sites do not have any control over the design of the protocol.  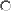 Yes 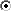 No |  |
| **6.8 OTHER SITE TYPES: Check all the other types of sites not affiliated with UCSF with which you are cooperating or collaborating on this project:** | |
| **Do NOT check any boxes below if this is a multi-center clinical trial, UCSF is just one of the sites, and neither UCSF nor one of its faculty-linked affiliates (SF VAMC, Gladstone, ZSFG) are the coordinating center.**  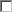 Other UC Campus 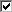 Other institution  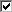 Other community-based site 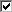 Foreign Country  List the foreign country/ies:  Rwanda  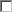 Sovereign Native American nation (e.g. Navajo Nation, Oglala Sioux Tribe, Havasupai, etc.) |  |
| **6.11 * OUTSIDE RELIANCES: (REQUIRED) Are any of the collaborating sites requesting to rely on UCSF's IRB:** | |
| 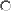 Yes 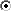 No |  |
| **6.14 * RELYING ON AN EXTERNAL IRB: (REQUIRED) Does this application include a request to rely on an external IRB (a central IRB (other than the NCI CIRB) or an external IRB (other UC campus, commercial, or institutional):** | |
| 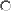 Yes No |  |
| **7.0 Outside Site Information** | |
| **7.1 Outside Site Information** | |
|  |  |

###### If you have more than 10 sites to add, list the outside sites in the Outside Sites List document and upload it in the Other Study Documents section of the Initial Review Submission Packet form. Any sites requesting to rely on UCSF's IRB must be listed below.

Click "Add a new row" to enter information for a site. Click it again to add a second site again to add a third site, a fourth site, etc.

| **Outside Site Information** | |
| --- | --- |
| **Non-UCSF affiliated site information:** | |
| Site name: University of Rwanda  Contact name:  Sabine Musange  Email:  [smusange@nursph.org](mailto:smusange@nursph.org) Phone:  +250788420378 |  |
| **For Federally-funded studies only, corresponding FWA#:** | |
|  |  |
| *** The research at this site will be reviewed by:** | |
| The non-affiliated site's IRB or a private IRB  The non-affiliated site is requesting UCSF to be the IRB of record for this study The non-affiliated site is not engaged in the human subjects research and has  provided a letter of support  If the other site's IRB approval letter is available now, attach it to the application. If the IRB approval letter is not yet available, submit it once you receive it.  Or, if the other site is [**not engaged**](http://irb.ucsf.edu/node/886#engaged) in human subjects research, attach the letter of support to your application. |  |

**Outside Site Information**

**Non-UCSF affiliated site information:**

Site name:

Rwanda Biomedical Center

|  |  |
| --- | --- |
| **8.0 Research Plan and Procedures** | |
| **8.1 HYPOTHESIS: Describe the hypothesis or what the study hopes to prove:** | |
| In Rwanda, PTBi East Africa is working in collaboration with the University of Rwanda and the Ministry of Health (MOH). The focus of this cluster randomized control trial is on antenatal care (ANC) and postnatal care (PNC) at the health center level. We hypothesize that group ANC/PNC implemented at the health center level will increase gestational age at birth among women who receive group care compared to women who receive standard focused ANC. To improve assessment of gestational age, we will also introduce ultrasound at the health center level for half of our facilities. These facilities will also incorporate pregnancy testing with urine dipstick to be performed by community health workers to facilitate early entry into ANC. |  |
| **8.2 AIMS: List the specific aims:** | |
| This cluster randomized control trial (RCT) aims to determine the effect of a group care model, which incorporates both group care ANC and PNC implemented at the health center level, on the following:   1. Gestational age (GA) at birth; 2. Adherence to the recommended four ANC visits; and |  |

|  | Contact name:  Felix Sayinzoga  Email:  [felix.sayinzoga@gmail.com](mailto:felix.sayinzoga@gmail.com) Phone:  +250788517814 |  |  |
| --- | --- | --- | --- |
|  | **For Federally-funded studies only, corresponding FWA#:** | |  |
|  |  |  |  |
|  | *** The research at this site will be reviewed by:** | |  |
|  | The non-affiliated site's IRB or a private IRB  The non-affiliated site is requesting UCSF to be the IRB of record for this study  The non-affiliated site is not engaged in the human subjects research and has provided a letter of support  If the other site's IRB approval letter is available now, attach it to the application. If the IRB approval letter is not yet available, submit it once you receive it.  Or, if the other site is [**not engaged**](http://irb.ucsf.edu/node/886#engaged) in human subjects research, attach the letter of support to your application. |  |  |
|  | | | |

| 3. Mortality at 42 days among preterm and low birthweight infants.  The primary objective is to assess the effectiveness of group ANC versus standard focused ANC in increasing GA at birth (among all babies).  Secondary objectives of this RCT are to answer the following questions:   1. Is there an effect of a group care model in reducing preterm birth rate? 2. Is there an effect of a group care model in increasing GA at birth among preterm births? 3. Is the effect of a group care model modified by the context of providing health center-level ultrasound services and community-level pregnancy testing? If so, how? 4. Do health centers providing group care show improved rates of adherence to the recommended four ANC visits? 5. Does a group care model mitigate select maternal morbidities, particularly reflected in the number of C-sections performed? 6. Does a group care model improve the 42-day outcomes, both in regards to mortality and select morbidities (to be determined), among all neonates and specifically among preterm neonates? |  |
| --- | --- |
| **8.3 DESIGN: Briefly describe the study design (e.g., observational, interventional, randomized, placebo- controlled, blinded, cross-over, cross-sectional, longitudinal, pharmacokinetic, etc.):** | |
| In this cluster RCT, we will pair-match facilities based on factors affecting GA at delivery among ANC enrollments, quality of ANC and PNC, potential lost-to-follow-up (LTFU) rate, and additional criteria pertaining to facility capacity. Within each pair, a facility will be randomly chosen to implement the intervention, a model of group ANC and PNC, while the control facility will continue to provide standard ANC and PNC. Pairs will then be further matched with other pairs into quadruples (to the extent possible), within which one pair will be assigned to additionally implement ultrasound at the health center level and early pregnancy testing at the community level. Overall, each facility will have one of the following assignments:  ***Arm 1*** : Standard ANC and PNC care only (pure control)  ***Arm 2*** : Standard ANC and PNC care, with the addition of early pregnancy testing and ultrasound  ***Arm 3*** : Group ANC and PNC only  ***Arm 4*** : Group ANC and PNC care, with the addition of early pregnancy testing and ultrasound  This design will enable us to conduct our primary analysis on the difference between group care and standard care, but also assess if that effect is mediated by early pregnancy testing in the community or the availability of ultrasound at the health center. |  |
| **8.4 BACKGROUND AND SIGNIFICANCE: Briefly provide the background and significance of this study (e.g. why is this study needed) (space limit: one half page):** | |
| Stillbirths and neonatal deaths can be prevented with appropriate and timely antenatal care (ANC). In Sub-Saharan  Africa, 69% of pregnant women receive at least one ANC visit and 44% receive at least four ANC visits (Lincetto WHO). However, the number of ANC visits does not provide information about the quality of care received.  Enhanced ANC, including the detection of pregnancy complications, has been modeled to prevent an estimated 21% of stillbirths (Bhutta 2014). However, there is limited data showing how improved quality ANC directly affects neonatal mortality, and more specifically, preterm birth rates (birth before 37 weeks of pregnancy). In Rwanda, 99% of pregnant women have attended at least one ANC visit with skilled medical personnel, while only 43.9% have attended the recommended four or more standards ANC visits. Among all |  |

| women ages 15-49 years who had given birth in the two preceding years, 41.6  % had received a PNC assessment in the first two days after birth (National Institute of Statistics of Rwanda, 2015).  An innovative method of delivering ANC in a group model has shown an impact on preterm birth rates in the United States. A randomized control trial (RCT) of a group ANC model, known as CenteringPregnancy (CP), demonstrated a 33% reduction in preterm birth rates among group prenatal care participants (Ickovics 2007). The CP model includes ten group antenatal sessions for 8-12 women of similar gestational age, with strong emphasis on facilitative leadership and women’s empowerment. A health provider facilitates group discussions based on the group’s needs and interest, and women learn how to measure their own weight and blood pressure. However, relatively few studies have been conducted for CP, and a recent Cochrane review reported that women in group ANC showed high levels of satisfaction, but showed no significant effect on preterm birth (Catling 2015).  CP has been implemented internationally, but mostly in high-income settings and at smaller scales (e.g. United States, Netherlands, and Australia). CP Netherlands is also currently testing expanding group care to postnatal and infant visits (Group Care HSPH 2014). CP-based group ANC has also been piloted in sub-Saharan Africa, and while feasibility and acceptability studies indicate that this model of delivery was well-received in Malawi and Tanzania, no results pertaining to newborn outcomes are available at this time. It should be noted that evidence from women’s participatory action groups have shown to positively influence health seeking behavior and result in a 23% reduction in neonatal mortality (increased to 33% reduction when at least 30% of pregnant women participate) (Prost 2013). Given the relatively high impact in the Ickovics trial and the paucity of other interventions with this degree of impact on preterm birth outcomes, further studies are warranted. Additionally, no studies have determined if gANC can be leveraged to increase PNC visits and other secondary outcomes.  PTBi East Africa aims to address the growing problem of prematurity in Rwanda with a focus on group ANC and PNC at the health center level in five districts.  Rwanda provides an excellent opportunity to test a group model with contributing factors including, but not limited to: community capacity; cultural foundations in community-based empowerment work; robust existing data systems including longitudinal ANC registers, etc. This infrastructure also allows for great potential for the early identification of pregnancy through community health worker networks and ultrasound at health center level, per needs identified by our partners. |  |
| --- | --- |
| **8.5 PRELIMINARY STUDIES: Briefly summarize any preliminary studies relevant to your proposed research (space limit: one half page):** | |
| Our group has not published any specific preliminary studies in this area; however, our group has conducted facility assessments to inform site selection and qualitative work to inform the group model development. Implementation and evaluation of the group ANC model has been studied in other settings. |  |
| **8.6 * TREATMENT PROTOCOL: Is this a treatment study, i.e. does this study intend to provide treatment to individuals with a medical or psychological condition: (REQUIRED)** | |
| Yes No |  |
| **8.7 * BILLABLE PROCEDURES: Does this study involve any procedures, lab tests or imaging studies that have a CPT code and could be billable to patients, their insurance, Medi-Cal, Medicare, or any other entity (answer 'Yes' even if the study is going to pay for all the procedures): (REQUIRED)** | |
| Yes No  **If you are not sure if your study involves billable procedures, send an email to the UCSF Office of Clinical Research (OCR) for help answering this question.** |  |

| **8.8 * COMMON RESEARCH ACTIVITIES: Types of research activities that will be carried out. Check all that apply and describe in more detail in the 'Procedures / Methods' section: (REQUIRED)** | |
| --- | --- |
| Interviews, questionnaires, surveys Educational or cognitive tests  Focus groups  Social media-based research activities Observation  Fitness tests or other exertion activities Use of mobile health apps or other apps  Collection of data from wearable tech such as Fitbit, Apple Watch, Garmin, motion actigraphs, etc.) Non-invasive imaging or testing (MRI, EEG, pulse oximetry, etc.)  Imaging procedures or treatment procedures that involve radiation (x-rays, CT scans, CT-guided biopsies, DEXA scans, MUGA or PET scan)  Administration of contrast agent  Randomization to one intervention versus another Use of placebo  Biopsy conducted solely for research purposes Sham surgical procedure  None of the above |  |
| **8.9 * PROCEDURES / METHODS: (REQUIRED)** | |
| Describe the research methods and study activities taking place at each site (e.g. what will participants be asked to do and what will members of the study team do?). If there will be multiple participant groups or study sites, explain what will happen with each group or study sites.  If some of the activities would occur even if the person were not in the study, as in the case of treatment or tests performed for diagnostic purposes,  **clearly differentiate between those activities that will be done solely for research purposes and those that are happening as part of routine care.**  Please call our office at 415-476-1814 and ask to speak to someone on the Expedited Review team if you need help differentiating between what parts are research and what parts aren't.  In facilities randomized to group care (Arms 3 and 4), this modality will be the opt-out standard of health facility-based ANC and PNC. Individual visits will continue to be offered for women and newborns requiring episodic examination and management, and for those who may decline group care participation.  When a pregnant woman presents at the health facility for her first ANC visit (ANC 1), she will experience the standard of care as described in the Rwanda Focused Antenatal Care Reference Manual (2015, p. 15- 16). At the conclusion of the ANC 1 visit, if ongoing ANC at the health facility is most appropriate for the pregnant mother, the provider will invite the woman to participate in group ANC/PNC and briefly describe it. Key messages delivered will be:  Group ANC/PNC offers connection and knowledge-sharing with other pregnant women Confidentiality is prioritized by all participants  You are part of a consistent group of women who will gather for each visit; this group of women are living the same experiences you are and are likely to have similar needs and questions  All health assessments, screening and treatments to promote a healthy pregnancy are delivered |  |

| All the dates and times of future ANC and the 6-week PNC visit will be given to participants at the time of ANC 1.  An important activity of ANC 1 is group assignment based on gestaional age (GA). After identifying the woman’s due date, the provider uses the health facility’s group ANC calendar to assign the woman to a group of 8-12 women with a similar due date (within the same 2-week period is preferred, within the same 4-week period is acceptable). Once the woman has been assigned to a group, all the dates of her group ANC(gANC) visits 2-4 and group PNC (gPNC) 6 weeks are known and the provider communicates these to her. Over the course of ANC, all women will be encouraged to give birth in a facility, where the first PNC visit is delivered, and to seek PNC care from the CHW when the newborn is 3 and 7 days old.  The first gANC visit is timed so that the woman returns between 18-22 weeks GA. The second gANC visit occurs between 26-30 weeks GA. The third gANC visit is between 34-38 weeks GA (36-38 weeks is ideal). This schedule places all group care visits 8 weeks apart and simplifies group scheduling for the health facility. Those attending ANC 1 late in pregnancy are invited to attend the remaining scheduled visits of their assigned group (by estimated due date) and are also invited to attend the visits they have missed (for example, gANC 2 and/or 3) with different groups. It is ideal for a group of women to be entirely consistent over time; however, it is practical to plan for some movement of women between groups as needed. The goal is that by 6 weeks after birth women will meet for a final group visit with other mothers they already know—with whom they have formed close connections during gANC.  The Rwanda gANC/PNC model was developed by the Technical Working Group, which is composed of representatives from maternal-child health stakeholder organizations in Rwanda. Other key characteristics of the Rwanda gANC/PNC model are:   1. Women sit in a circle in a group space where other staff and patients do not enter during the visit. 2. Two co-facilitators lead each group visit: one ANC provider (midwife/nurse) and one ASM (Agent de Sante Maternelle, maternal health volunteer). 3. Confidentiality and mutual respect are prioritized by pregnant women and co-facilitators. 4. Clean water is offered to the women to drink while they socialize during the first 30 minutes. 5. Health assessments are conducted on a rolling basis during the first 30 minutes, as women arrive at the scheduled visit time. 6. Women participate in their own health assessments as much as possible (blood pressure and weight). 7. Consultations are conducted in a semi-private area of the group space. 8. Women and babies receive the routine assessments, screening, and treatments described in the Rwanda ANC/PNC packages, as well as treatments indicated for special conditions. 9. Women are referred to the district hospital for abnormal conditions, according to current national guidelines. The doctor with whom a referred woman consults will develop a plan of care and indicate on the counter-referral form whether or not she should continue to attend ANC visits at the health facility. 10. Key messages consistent with Rwanda’s ANC and PNC packages are delivered through facilitated discussion in which the women speak more than the facilitators. 11. Learning activities are based on principles of adult education, including repetition, peer-to- peer teaching, engagement of several different senses, and fun. 12. Each group of women decides if they will invite husbands and next-of-kin to attend group visits. 13. Group discussion begins after health assessments are completed and lasts 1 hour. |  |
| --- | --- |

| 1. Co-facilitators “debrief” after every group visit in a continuous learning and quality improvement process. 2. Women are invited to return to the health facility at any time for individual episodic evaluation of danger signs or any other concerns.   **Facilitation**  The success of the group care model relies on excellent facilitation skills employed by the providers and ASMs who conduct group visits. A facilitator is defined as a person who helps a group improve the way it identifies and solves problems and makes decisions. In the gANC/PNC model one midwife or nurse and one ASM act as co-facilitators, guiding the group through content (key messages) and process (how topics are discussed). Facilitation will be a new skill set for many midwives, nurses, and ASMs who may be accustomed to providing health care and health information in a hierarchical, didactic manner.  The facilitators’ main tasks are to:  Promote knowledge-sharing between group members—because people are more likely to change their behavior with encouragement from a peer than instruction from an authority;  Encourage group members to make their own choices and be accountable for them— because while external controls generate compliance, internal controls generate commitment and empowerment; and  Model behaviors she/he hopes group members will adopt—such as self- awareness, self-care, compassion, and respect; and  Share relevant information as the content expert when knowledge gaps exist.  An effective facilitator enables the group process to the degree that group participants speak more during the discussion than the facilitator(s) speaks.  Once health facilities have been randomized, those assigned to group care (Arms 3 and 4) will each choose 3 providers to be trained in the gANC/PNC model, including facilitation. 12 ASMs per health facility will also be chosen to receive the same training, as equals and partners to the providers. Each new facilitator will attend a three-day training course in which they will learn how to implement all the key components of the group care model—including learning how to facilitate group discussions.  New facilitators require practice and feedback from expert facilitators in order to gradually improve their efficacy. For this purpose, a small group of Rwandan “Master Trainers” will be selected (based on their existing skills and related experience) and prepared with detailed training in group care and facilitation. These 6-8 trainers will, under the direction of the Principal Investigators, conduct site visits at health facilities providing the group care intervention. These visits will be frequent immediately after implementation and then intermittent over the course of the trial. During these site visits, Master Trainers will monitor model fidelity, observe facilitators as they provide group care, and offer feedback and/or re- training using their own advanced facilitation skills. The gANC/PNC *Model Fidelity Assessment* will be used to guide the Master Trainer’s supportive activities during the site visit. The results of these skills assessments will be monitored for changes over time.  Facilitators will be invited to ask for additional implementation support and re-training as they need it. After every group visit, co-facilitators participate in a short “debrief” process in which they thoughtfully consider what went well during the group visit and what could be improved to make the next group visit even more effective. During this debrief, the co-facilitators enter their responses into a RedCap platform using a tablet provided for this purpose. The final question of this *Group Visit Debrief Questionnaire* asks if the co-facilitators feel they need implementation assistance or more training. If they answer in the affirmative, the Program Manager will arrange for a Master Trainer to make additional site visits as needed. During the facilitators’ two-day training course, they will be trained on how to use this RedCap debrief tool and consented so that any relevant process or implementation data can be used for analysis.  **Implementation**  While each health facility randomized to provide group ANC/PNC will create its own unique implementation plan based on the days of the week ANC is provided and the number of ANC providers available on those days, all health facilities will implement group care following the same general guidelines:  For every 8-12 women due each month at the health facility, a new group will be organized. This group is scheduled to meet every 8 weeks over the course of 24 weeks, for four total group visits. |  |
| --- | --- |

| The number of groups formed sharing the same due month will equal the number of group visits held per week at the health facility.  Group ANC/PNC will be implemented across all 18 health facilities in a “phased” manner. Implementation is phased in two ways:   1. *Across health facilities*. Health facilities will be divided into three groups of six. Six health facilities will implement simultaneously, six more will implement two weeks later, and the final six will implement two weeks later. This phased roll-out allows the six Master Trainers to each support one health facility during the first two weeks of implementation. Over the course of six weeks, each Master Trainer supports three health facilities that implement in sequence at two- week intervals. 2. *Within each health facility* . Each health facility will not change ANC operations from individual to group care all at once. Transitioning from status quo to universal group care at the facility will require 24 weeks. This transition will proceed as follows:    1. Once the health facility has set its “group care start date,” 18 weeks are added to this date. This is the pregnancy due date used to enroll women into group care during the initial 24 weeks of the trial.    2. Women with a pregnancy due date on or after this date are invited to attend group care, oriented, and enrolled—all during ANC 1.    3. Women with pregnancy due dates before this date continue to receive standard, individual care throughout pregnancy and the postnatal period. This is true ONLY during the first 24 weeks of the trial, until the phased implementation of group care at the facility is complete.    4. The first group care delivered at the health facility will be for women with this due date plus 2-4 weeks. These women meet on the “group care start date” for gANC 2.    5. Health facilities can calculate how many group visits per week they must plan in order to accommodate all women seeking ANC at steady state (24 weeks after trial start) using the following formula:   *# of women due per month* ***divided by*** *# of women per group (maximum 12)*  For example, a health facility that expects an average of 48 women to give birth per month will at steady state offer 4 group visits per week. 75% of these group visits are ANC and 25% of these group visits are PNC, at steady state.   - 1. Health facilities will start with—on average—one group visit per week and slowly work up to steady state by 24 weeks after implementation.   Of the selected 18 pairs of health centers, 9 pairs will additionally be assigned to conduct early pregnancy testing at the community level and first-trimester ultrasound at the health center level. Consequently, 9 of the sites providing these additional services will be simultaneously conducting group care (Arm 4), whereas the additional nine will be conducting standard ANC and PNC (Arm 2).  **Ultrasound**  The objective of ANC1 screening ultrasound (at late 1st or early 2nd trimester) at the health center level is to accurately assess GA and identify any fetal malformations. Approximately 2 ANC providers per health center will be trained to use and conduct ultrasound. We adapted first- trimester components of the University of Washington ultrasound curriculum in order to address the needs of the local context.  The Rwanda Radiology Society was contracted to conduct ultrasound training and continuous mentorship, under the supervision of Dr. Felix Sayinzoga, Maternal Child and Community Health Division Manager. The Rwanda Radiologist key trainers will undergo a 3-day training of trainers in Kigali to align ultrasound activities and objectives to the scope of the manufacturer’s training manuals. Training objectives and outcomes will be jointly defined and the mentorship/quality assessment tools will be created and customized to fit local context relevance in collaboration with the manufacturer’s team.  In order to train providers, we will contact health centers and ask them to select 3 ANC providers for training. Two rounds of trainings will be conducted in Bugesera, each led by a team of 4 Radiologists and with 18 participants. The training will last 10 days and will include an introduction to theory and an important component of practice in surrounding health centers.  Ultrasound devices will provide the option of saving images/videos that can be shared via the Internet or a USB. The Radiology Society will provide guidance on the creation of a platform for assessing images and providing feedback to ANC providers. The society will engage in regular onsite mentorship visits across health centers. The Rwanda Radiologist key trainers will conduct 2 visits per month per health center during the first 3 months of the trial to ensure close mentorship and quality assessment of the ultrasound exams conducted by the nurses/midwives during the first months of implementation. |  |
| --- | --- |

| Moreover, radiologist technicians in each district will routinely conduct spot checks in facilities, and 1 main radiologist will act as the supervisor and conduct image quality control. Each facility will receive quarterly visit unless the health center’s team expresses the need for more assistance. In this case, the Program Manager will arrange for a Rwanda Radiologist key trainer to make additional visits as needed.  **Early pregnancy testing**  In order to promote early entry into ANC, CHWs will conduct urine pregnancy testing among women in communities. The aims of urine pregnancy testing are to:  Aid women in the early detection of pregnancy in order to engage them in early ANC Confirm pregnancy by detecting human chorionic gonadotrophic hormone (hCG) in urine Promote early referral of at risk pregnancies  We will need approximately 25,000 urine pregnancy testing kits annually for 18 health centers. This figure was determined by multiplying the DHIS monthly ANC1 average by 1.25. This accounts for more tests than positives. We will procure testing kits through the district pharmacies to ensure that health centers can distribute adequate testing kits to CHWs.  A participant and user manual will be developed by a consultant under the supervision of Catherine Mugeni and Dr. Felix Sayinzoga of the Rwanda Biomedical Center Maternal Child and Community Health Division. These manuals will be available both in English and in Kinyarwanda and inserted into the national curriculum for maternal and newborn health.  Health care providers will conduct training in urine pregnancy testing for CHW supervisors and ASMs from catchment areas surrounding their respective health center.  CHW supervisors at health centers will provide ongoing monitoring and supervision of ASMs and will report to health care providers on a monthly basis. ASMs will report all new pregnancies using Rapid SMS and women will be responsible for submitting their urine pregnancy test results to their nearest health center.  **Procedures at enrollment**  At intervention sites, enrollment of participants will be completed by field coordinators hired and trained in group care, in how to conduct enrollment and administer consent. Only women attending ANC for the first time will be offered group care. Enrollment processes will include entering participant contact detail, medical history in the *enrollment form* and completion of the consent process by agreeing or disagreeing to the consent statement read by the participant herself or by a witness if the participant is illiterate. The consent statement will explain the study objectives, requirements, potential risks, privacy and ethical obligations of the research team. This will be followed by a second stage consent process which requires participants to demonstrate their understanding of the nature and requirements of the research including understanding that participation is voluntary and that information is kept private.  Participants who are unable to provide consent or who are unwell during this first visit may be re-offered to enroll in group care at their next visit, if they still meet the eligibility criteria.  At control sites, participants were initially enrolled by the health facility personnel, as they are consenting only to the ongoing collection of data and nothing in their care is changing. Effective January 2018, we have added additional field coordinators so that control sites also have a designated field coordinator to enroll women and collect data. Enrollment processes will include entering participant contact detail, medical history in the enrollment form and completion of the consent process by agreeing or disagreeing to the consent statement read by the participant herself or by a witness if the participant is illiterate. The consent statement will explain the study objectives, requirements, potential risks, privacy and ethical obligations of the research team. This will be followed by a second stage consent process which requires participants to demonstrate their understanding of the nature and requirements of the research including understanding that participation is voluntary and that information is kept private.  Participants who are unable to provide consent or who are unwell during this first visit may be consented on a subsequent visit.  This project has received local permission to waive parental consent for pregnant minors ages 15 and older. Pregnant minors younger than age 15 will still require parental assent to participate. To date we have not had contact with any pregnant minors younger than age 15.  **Follow-up of cohorts**  Each health center providing group care will be assigned one “field coordinator” who will also serve as the data collector for that site and one nearby control site. The data collectors will be responsible for following up on participants at the health center where they are based, and he/she will also be expected to routinely visit the corresponding control site to track the participants enrolled there. Effective January 2018, we are expanding the number of field coordinator to be 1 per site. |  |
| --- | --- |

| All women enrolled in the study will be followed across their pregnancy, delivery, and up to 42 days post- delivery. Women will be grouped into cohorts based on entry into ANC. We will be using existing national data collection tools (ANC/PNC patient files, ANC registers, Maternity/L&D registers, PNC and neonatal registers) as a data source. Each section below provides an illustrative list of the data elements to be collected at each stage. To see a full list of data elements being collected for each participant.  *Enrollment at ANC1*  Each participant will complete an *Enrollment form* asking a few key questions to identify her and assess key covariates for pregnancy outcomes including SES, indoor air pollution, and food security and anxiety. Next she will be assigned a unique study ID, which helps to serve as the linking mechanism across exposure period of interest. In order to assign a study ID and to track the progression and outcome of the pregnancy, we will request to collect names, national IDs, and phone numbers to facilitate longitudinal tracking and linkage across different data sources and service delivery wards (both within and across facilities). We will additionally collect data from the ANC register and ANC/PNC  file around demographics and key obstetric history (age, parity, gravidity, Last Menstrual Period,  Expected Date of Delivery, etc.) We will also request consent for follow-up through (1) tracking through registries and medical files; (2) contacting participants via phone; (3) and/or contacting community health workers.  Due to the absence of a linking identifier in facility registers—namely the ANC, maternity, neonatal, and PNC registers—we will be using a combination of name, date of birth, village, and gravidity/parity (where available) to track a participant. National ID will allow us to link information from the facility registers with RapidSMS, particularly in cases where a woman is lost-to-follow-up or information on delivery or infant outcomes is confined to the community level.  As each pregnant woman is enrolled into the study, her information will be collected using a tablet-based data collection tool, saved into a cohort database, and uploaded onto a secure server. Any unique identifiers will be kept confidential and will only be used for purposes of linking data between different sources. Once a participant has been followed up through the full 42-days post-delivery, study staff will remove all personal identifiers from the analysis data set. A separate secure dataset will be kept with all linking data and will be accessible to the local field team for tracking purposes only. The study ID will be the only remaining case identifier in the analysis data set and will only be known to the study team.  *Additional Antenatal Care Visits*  Through data strengthening, facility staff will receive training to better document specific data elements of interest in the antenatal register, including gestational age at each visit, identified risk factors, referrals, additional ANC visits, and pregnancy outcomes. On a routine basis, data collectors will go through facility antenatal registers and ANC/PNC files to identify mothers who have returned for their subsequent ANC visits (ANC2, ANC3, and ANC4). In addition, data collectors will also note (1) which mothers may have delivered early, and (2) of any deliveries, were there complications and how were these complications managed, (i.e., was the case identified and referred to the district hospital; was the case identified and handled at the health center, etc.). With gANC implementation, we anticipate an increase in additional ANC visits (>4 visits); data collectors will be trained to abstract these additional ANC visits as well. Last but not least, data collectors will also record any pregnancy outcomes as noted in the antenatal register.  In addition to collecting register elements, field coordinators will review patient files of all enrolled pregnant women to document data on the process of care which will allow us to more deeply compare the models. They will document such variables as obstetric history, weight, blood pressure fundal height, diagnosis or treatment of malaria or STIs, identification of hypertension, preeclampsia or other risk factors. These data will allow us to analyze whether women receive substantially the same care in the two arms and whether there is any variance in the quality of care across arms or individual centers.  The number of women who experienced referral to the health center for ANC after a positive pregnancy test completed in the community will be captured by abstracting data from the UPT referral form developed by the Rwanda Ministry of Health. The findings and some process measures documented during ultrasound examinations completed by ANC providers will be abstracted from the Ultrasound Report Form, which was co-developed with the Rwanda Society of Radiologists for this trial.  *Delivery: Normal and Complicated Cases*  On a routine basis, on-site data collectors will abstract pertinent data elements from the facility maternity register. Ideally, mothers who attend ANC sessions at the health center will also deliver at the same location. In this case, a data collector will track these mothers using name, village, and gravidity/parity in the maternity register and abstract the delivery information (e.g., date of delivery, mode of delivery, complications, term/preterm); and newborn information (e.g., sex, weight, APGAR score at 1minute, head circumference, length, birth weight, chest circumference, foot length, and mid-upper arm circumference). |  |
| --- | --- |

In cases where a mother had a complication and was referred up to the district hospital, data staff will track these referred cases at the referral hospital. The data collector will abstract the same information from the maternity register as was collected for women who delivered at the health center level. If newborns were transferred to the neonatal ward/unit after birth, data staff will also identify newborn outcomes using the facility neonatal register.

*Postnatal Care & Infant Outcomes*

Using the *Participant Tracking Tool*, data staff will be reminded of the six-week post-delivery date. National guidelines state that postnatal sessions will be conducted at the original health center in which a woman attended her ANC visits. It is expected that women who (a) delivered at the health center, or (b) delivered at the district hospital and were discharged will attend PNC at the health center. Data staff will abstract data elements from the facility postnatal register, including date of PNC visit, certain anthropometric newborn measures (weight, length, head circumference at consultation), and mother information (MUAC, Vitamin A). For mothers who had complicated deliveries and needed additional monitoring at the district hospital, postnatal information on the newborn may be housed at the district hospital. In this case, the data staff will abstract the necessary data elements from the facility neonatal register.

*Lost-to-Follow-Up (LTFU) Cases*

Data collectors are responsible for monitoring the cohorts on a routine basis, and routine tracking should alert data staff to mothers and/or infants who are LTFU (i.e., did not attend additional ANC, delivered at a different location, did not attend PNC session). Participants who are LTFU will first be tracked using RapidSMS (a national real-time reporting and alert system which allows interactive communication between the ASM, health center, and the national centralized database) to determine their “true outcome” (dead, declined to continue with study, transferred out to a different facility, etc.) Data elements of interest to be abstracted from RapidSMS report include birth and death events, newborn care events, and any Red Alert events. These true outcomes, which are generated by the Data Manager at each health center, will be abstracted and updated in the cohort database by the data collector, to allow for analysis. If follow-up via RapidSMS is unsuccessful, study staff (trained nurses or data collectors) will contact the mother via telephone to query the health status of her child. Should we be unable to contact the subject, we will ask the CHW who works in that subject's village to help the research team obtain information about where the woman gave birth.

Surveys (Women participants and providers)

Other secondary outcomes will be measured using data collected through questionnaires administered to ANC and PNC participants. Immediately after giving consent for data analysis, a convenience sub-sample of women in all trial arms will be invited to participate in a baseline questionnaire. Consent (Appendix 6- 8a) will be obtained from women participating in both the baseline and 8-week follow-up questionnaires using the same form. The *Baseline Participant Survey* (Appendix 6) will measure locus of control, social support, pregnancy-related anxiety, and antenatal/postnatal knowledge. We will recruit for 12 months, 5 women per month per facility, on the first day ANC1 is offered each month at the health facility. A total of 2160 women will be recruited for the baseline survey, with 1080 in each arm.

Any enrolled woman who presents to the health center for immunization or postnatal care services between 4-12 weeks after birth is invited to participate in the *8-week Follow-up Participant Questionnaire*. This postnatal questionnaire will repeat the same set of questions asked at baseline about locus of control, social support, pregnancy-related anxiety, and antenatal/postnatal knowledge and will also ask each woman to report whether or not she is currently using a contraceptive method and, if so, which method. This questionnaire has been tailored for women receiving standard ANC/PNC care (Appendix 7) and women receiving GANC/PNC (Appendix 8). We will recruit a total of 1080 women to participate in the postnatal questionnaire, or 540 in each arm. Assuming a baseline family planning rate of 45%, with this sample size we will be able to detect a 20% increase in family planning at 80% power and 5% significance.

The respondents to the *Baseline Participant Survey* and the *8-week Follow-up Participant Questionnaire* will be unmatched cohorts, as we have found follow-up of a matched cohort to not be feasible in this context.

Characteristics and job satisfaction of ANC and PNC providers will be measured via the *Baseline Provider Questionnaire* administered at the initiation of the trial. It will ask about:

Basic characteristics such as age, professional title, and years of experience; Job satisfaction;

Motivation, beliefs about patient-centered care, and temperament; and Perceived Stress.

The *Follow-up Provider Questionnaire* will be administered to the same sample of providers at 9 months and 18 months after the trial begins, with questions tailored specifically to standard care and group care providers, respectively.

Standard care providers are asked the same set of questions

| about job satisfaction as were asked at baseline. Group care providers are also asked these same questions about job satisfaction, with additional queries about whether they prefer to deliver group ANC or PNC versus standard ANC or PNC. As this questionnaire will be administered longitudinally, any inter- or intra- provider differences in job satisfaction will be reported.  **Focus Group Discussions (Women and Providers in Group Care)**  Qualitative work with women and providers to inform any fine tuning to the model will be conducted between 9 and 18 months after implementation. Four to eight focus groups for women, 4-6 focus groups for providers, and 2-4 focus groups for community health workers will be held to get in-depth insight into their perceptions of the model.  *Cost-effectiveness data* will be collected in order to evaluate the relative cost and effectiveness of the two models. Overall trial outcomes will be used for the effectiveness data but costs data will be collected to assess the cost of each type of care. |  |
| --- | --- |
| **8.11 INSTRUMENTS: List all questionnaires, surveys, interview, or focus group guides that will be used for this study:** | |
| If the instruments are not complete or not available because they will be developed as part of this study, describe the basic content or include an outline and submit the final versions to the IRB with a modification for approval prior to use.  Appendix T Ultrasound Report Form  Appendix U UPT Referral Form  Appendix 12 Participant Focus Group Discussion Guide, mid-point Appendix 13 Provider Focus Group Discussion Guide, end-point  We will develop a tool to collect cost information so that we can assess cost-effectiveness, but that will be available only after we have launched the model and have received feedback about any suggested changes in the implementation approach. Our team is working directly with the head of maternal and child health at the Rwanda Biomedical Center (the implementing arm of MOH) and thus well positioned to get this information most effectively once the initial launch is complete.  **Attach any unpublished instruments in the 'Other Study Documents' section of the Initial Review Submission Packet form after completing the study application. Published instruments should NOT be attached.** |  |
| **8.12 * BIOSPECIMEN COLLECTION: Are you drawing any blood or collecting other biosamples (e.g. tissue, buccal swabs, urine, saliva, hair, etc.) for analysis under this protocol and/or storage for future research: (REQUIRED)** | |

| Appendix A | Model Fidelity Assessment |
| --- | --- |
| Appendix B | Group Visit Debrief Questionnaire |
| Appendix C | Enrollment Form |
| Appendix D | ANC Register: Initial Visit |
| Appendix E | ANC/PNC: Initial Visit |
| Appendix F | ANC Register: Follow up Visits |
| Appendix G | ANC/PNC: Follow up Visits |
| Appendix H | Maternity/L&D Register |
| Appendix I | Neonatal Register |
| Appendix J | Postnatal Register |
| Appendix K | Rapid SMS Report |
| Appendix L | Ultrasound Tool |
| Appendix M | Baseline Participant Questionnaire |
| Appendix N | 8-Week Postnatal Participant Questionnaire: Control |
| Appendix O | 8-Week Postnatal Participant Questionnaire: Group Care |
| Appendix P | Baseline Provider Questionnaire |
| Appendix Q | Follow-up Provider Questionnaire: Control |
| Appendix R | Follow-up Provider Questionnaire: Group Care |
| Appendix S | Tracking Tool |

| Yes No |  |
| --- | --- |
| **8.13 STATISTICAL METHODS: Briefly summarize the methods and types of analyses that will be performed:** | |
| We will compare facility-level Intention-to-treat average GA at delivery among women attending ANC in the group vs standard ANC facility. We will do this using a two-stage estimator, based on targeted maximum likelihood estimation. At the first stage, we will obtain a point-estimate of the facility-level average GA through an analysis of each facility’s individual-level data, adjusting for individual-level covariates to account for potential selection bias due to LTFU (e.g. women who are referred up for complications or drop out of care are more likely to be LTFU and also more likely to have delivered early). At a second stage, we will assess the intervention-effect by comparing the facility-level average GA between intervention and control sites, adjusting for facility-level covariates and accounting for pair clustering in variance estimation. |  |
| **8.14 REFERENCES: List only the 5-10 most relevant references (a separate bibliography can be attached for reference purposes if this study involves novel approaches, agents, or an emerging technology that the IRB may not be familiar with):** | |
| 1. Lincetto O, Mothebesoane-Anoh S, Gomez P, Munjanja S. Antenatal care. Chapter 2. Opportunities for Africa’s Newborns. 2. Bhutta, Z., Das, J., Bahl, R., Lawn, J., Salam, R., Paul, V., Walker, N. (2014). Can available interventions end preventable deaths in mothers, newborn babies, and stillbirths, and at what cost? *The Lancet, 384*(9940), 347-370. doi:10.1016/S0140-6736(14)60792-3 3. National Institute of Statistics of Rwanda. (2015). Demographic and Health Survey 2014-2015: Key Findings. 4. Ickovics, J., Kershaw, T., Westdahl, C., Magriples, U., Massey, Z., Reynolds, H., & Lawn, J. (2007). fGroup Prenatal Care and Perinatal Outcomes: A Randomized Controlled Trial. *Obstetrics & Gynecology, 110*(4), 993-994. doi:10.1097/01.AOG.0000275284.24298.23 5. Catling, C.J., Medley, N., Foureur, M., Ryan, C., Leap, N., Teate, A., Homer, CSE. Group versus conventional antenatal care for women. *Cochrane Database of Systematic Reviews* 2015, Issue 2. Art. No.: CD007622. doi: 10.1002/14651858.CD007622.pub3. 6. Maternal Health Task Force, Harvard School of Public Health, et al. Group Care. Innovative Methods 2014. 7. Prost A, et al. Women’s groups practising participatory learning and action to improve maternal and newborn health in low-resource settings: a systematic review and meta-analysis. Lancet. 2013 May 18; 381(9879): 1736–1746. |  |
| **9.0 Drugs and Devices** | |
| **9.1 * DRUGS AND/OR BIOLOGICS: Are you STUDYING any drugs and/or biologics that are either approved or unapproved: (REQUIRED)** | |
| Yes No |  |
| **9.3 * MEDICAL DEVICES: Are you STUDYING any medical devices, in vitro diagnostics, or assays that are either approved or unapproved:(REQUIRED)** | |
|  |  |

| Yes No |  |
| --- | --- |
| **9.4 * NSR: Are you requesting a Non-Significant Risk (NSR) determination for an investigational device: (REQUIRED) Note: an** [**NSR determination**](http://irb.ucsf.edu/node/726) **is different from an Investigational Device Exemption (IDE). Check the Help link for more guidance on what types of devices can qualify for an NSR determination.** | |
| Yes No |  |
| **9.5 LIST THE DEVICES: List the medical devices or in vitro diagnostics to be studied or used. In the device details screen you will be asked questions such as:**  **Whether the device is FDA approved or investigational Medicare device category**  **If the device will be provided at no cost**  **If an IDE is necessary, the IDE number, and who holds the IDE Risk category of the device**  **FDA status of the device**  **Please see the** [**UCSF IRB website**](http://irb.ucsf.edu/node/721#not) **for more details about the use of devices in research, including the** [**Investigator Checklist for Significant Risk, Non-Significant Risk, and/or IDE Exempt Device Studies**](http://irb.ucsf.edu/sites/hrpp.ucsf.edu/files/device-checklist.pdf) **Verification of IDE numbers: If the sponsor’s protocol does not list the IDE number, you must submit documentation from the sponsor or FDA identifying the IDE number for this study. Attach this documentation in the Other Study Documents section of the Initial Review Submission Packet. If you have any correspondence from the FDA or sponsor regarding this device, please attach it to the application.** | |
|  |  |
| **9.6 * EXPANDED ACCESS: Is this an expanded access or compassionate use protocol, meaning the primary purpose is to diagnose, monitor or treat a patient's condition, rather than the collection of safety and efficacy data of the experimental agent: (REQUIRED)** | |
|  |  |

| **View Details** | **Device Name** | | **Is the Device FDA Approved** | **Is this a new device or a new use of an already approved device** | **IDE Number** |
| --- | --- | --- | --- | --- | --- |
|  | Siemens Acuson P500 | | Yes | No |  |
| Manufacturer/Supplier of Device | | Siemens | | | |
| Medicare Category | | A B | | | |
| Where will the Devices Be Stored | |  | | | |
| Will Devices be supplied at no Cost | | No | | | |
| Is this a HUD (HDE) | | No | | | |
| HDE Number | |  | | | |
| Is the Device FDA Approved | | Yes | | | |
| Is this a new device or a new use of an already approved device | | No | | | |
| Is an IDE necessary | | No | | | |
| IDE Number | |  | | | |
| Who holds the IDE | | N/A | | | |
| IDE Details | |  | | | |
| In the opinion of the sponsor, select the level of risk associated with this device | | No Significant Risk | | | |

| Yes No |  |
| --- | --- |
| **10.0 Sample Size and Eligibility Criteria** | |
| **10.1 ENROLLMENT TARGET: How many people will you enroll:** | |
| 34,555  If there are multiple participant groups, indicate how many people will be in each group:  Outcome data for analysis or primary outcome: 214 woman-newborn pairs with complete ANC and birth outcome data, these women must have enrolled for ANC before 24 completed weeks gestation and attended at least 2 ANC visits during pregnancy. An assumption of a 30% loss-to-follow-up rate means the number of required enrollees at each facility, that meet these inclusion criteria, are 306  /facility x 36 facilities = 11,016. Our original application stated our enrollment target for pregnant women was 11,016.  We have revised the proposed enrollment number because: 1) data show only 50% of enrolled women return for at least 1 additional ANC visit (to date), 2) data show that approximately 65% of pregnant women present for ANC by 24 completed weeks of pregnancy, and 3) Rwandan partners decided to enroll all women at the study sites regardless of gestational age at enrollment. Our revised estimate is that we will need a maximum of 950 enrolled women per site to reach our target of 2 14 woman-newborn pairs eligible for the primary analysis. This brings our projected maximum enrollment to 34,200 pregnant women. Effective December 21, 2018, we have discontinued enrollment at 24,272 pregnant women in order to ensure we are able to follow-up all women to final outcome by study close.  Participant surveys: Previously, our enrollment target was 1080 women (sub set of outcome participants). However, we increased our recruitment goals regarding the participant survey from 1080 to 2160. We expect the loss-to-follow-up rate to be up to 50% due to the difficulty of persuading women, without incentives, to complete a follow-up questionnaire 4-8 week after birth, which is why we increased the recruitment numbers. We still hope to reach a sample size of 1080 postnatal surveys, but it seems unlikely as data collectors find it very difficult to meet women in person at the health center after birth.  Community Health Workers: 217 trained to be group care facilitators (group care facilitators consented for observation, debrief and a subset in focus groups)  Providers (Group care): 84 -180 providers (Group care facilitators, consented for observation, debrief, provider survey, and a subset in focus groups).  Providers (Standard care): 54 providers (consented for survey, subset in focus groups)  The combined number of group and standard care providers has been adjusted to include more providers because some providers originally trained (54 in the original cohort) were no longer available so new providers (180 (up to 10 more providers at each of 18 group care sites)) were trained and have been consented for relevant data collection activities. |  |
| **10.3 SAMPLE SIZE JUSTIFICATION: Explain how and why the number of people was chosen. For multi-site studies, this is referring to the number that will be enrolled across all sites:** | |
| *Group and Standard Care: Women*  We performed sample size calculations for a cluster RCT in which pair-matched facilities are randomly assigned to either provide group care or standard care. Based on the 2015 Cochrane Review (Catling 2015), we assumed the intraclass correlation coefficient (ICC) between both arms is no larger than  0.01. We assumed the standard deviation of GA at delivery is no larger than 4.3 weeks under both intervention and control arms. At 5% significance, with 36 facilities we are powered (80%) to detect a  0.5 week difference in GA at delivery with 214 observations (ANC+outcome) per facility. Assuming a follow-up rate of 70%, we would need to recruit 306 women per facility. At an average rate of 23 eligible recruits per month per facility, the total study duration would be (a) 14 recruitment months, (b) an additional six to seven months to observe the outcomes of the last cohort of women recruited, and  (c) three additional months to complete data processing and analysis.  We have revised the proposed enrollment number because: 1) data show only 50% of enrolled women return for at least 1 additional ANC visit (to date), 2) data show that approximately 65% of pregnant women present for ANC by 24 completed weeks of pregnancy, and 3) Rwandan partners decided to enroll all women at the study sites regardless of gestational age at enrollment. Our revised estimate is that we will need a maximum of 950 enrolled women per site to reach our target |  |

| of 2 14 woman-newborn pairs eligible for the primary analysis. This brings our projected maximum enrollment to 34,200 pregnant women.  *Group and Standard Care (Participant's Surveys)*: We will recruit for 15 months, 2 women per months per facility, through phone call surveys. This will be a total of 1080 women recruited, with 540 in each arm. A woman will be called after enrollment and 8 weeks after delivery to be administered the surveys. Allowing for a 10% loss to follow up rate, and assuming a baseline family planning rate of 45%, we will be able to detect a 20% increase in family planning at 80% power and 5% significance.  However, we increased our recruitment goals regarding the participant survey from 1080 to 2160. We expect the loss-to- follow-up rate to be up to 50% due to the difficulty of persuading women, without incentives, to complete a follow-up questionnaire 4-8 week after birth, which is why we increased the recruitment numbers.  Group and Standard Care: Providers  In our assessment of health centers and feasibility of group ANC/PNC in Rwanda, we identified that each health center in our study has a core team of 3-4 health care providers who are the “go-to” nurses or midwives that usually offer maternal and child health services. However, all providers at the health center have to float across service units as needed and as assigned by the head of health center. With a health center sample size of 18 for intervention and 18 for control, a core team of 54-84 providers would offer group ANC/PNC in intervention sites and 54-84 providers would offer standard care in control sites. As staff at health centers may turn over during the study period, and facilities may face unexpected changes  in the numbers of providers and allocation needs, it may be necessary to train more providers in group ANC  /PNC to maintain the integrity of the intervention. As health centers request that more providers be trained in group care, we will collaborate with heads of health centers to facilitate ongoing training and mentorship.  Group care Focus groups (Women): A convenience sample of 48-72 women in 6 focus groups will be asked to participate in focus group discussions after they give birth. These women would have been enrolled for the study at sites randomized to group ANC/PNC and ultrasound examinations.  Group care and or ultrasound providers Focus group (Providers and Community Health Workers): Providers offering group ANC/PNC will be asked to participate in focus group discussions. We expect that 48-72 providers and CHWs in 6 focus groups will participate.  Group and Standard Care (Provider Surveys): As mentioned above, we will enroll at least 54 providers in intervention sites and in control sites in a longitudinal survey We may enroll up to 10 more providers at each group care site if the heads of health centers request that we train more providers in group ANC/PNC.  Observations and debriefs of Group Care Providers and Community Health Workers: Master Trainers will observe providers in all intervention health centers to ascertain group care model fidelity. We expect that between 54-180 providers, and up to 217 CHWs will be observed. |  |
| --- | --- |
| **10.4 * PARTICIPANT AGE RANGE: Eligible age ranges: (REQUIRED)** | |
| 0-6 years  7-12 years  13-17 years  18-64 years  65+ |  |
| **10.5 * STUDY POPULATIONS: Data will be collected from or about the following types of people (check all that apply): (REQUIRED)** | |
| Inpatients Outpatients  Family members or caregivers Providers  People who have a condition but who are not being seen as patients Healthy volunteers  Students |  |

| Staff of UCSF or affiliated institutions None of the above |  |
| --- | --- |
| **10.6 * SPECIAL SUBJECT GROUPS: Check the populations that may be enrolled: (REQUIRED)** | |
| Children / Minors  Adult subjects unable to consent for themselves  Adult subjects unable to consent for themselves (emergency setting) Subjects with diminished capacity to consent  Subjects unable to read, speak or understand English Pregnant women  Fetuses Neonates Prisoners  Economically or educationally disadvantaged persons None of the above  If not already addressed in the Background and Significance questions in the Research Plan section or elsewhere, explain why it is appropriate to include the types of subjects checked above in this particular study:  At both intervention and control facilities, all pregnant women who enroll in ANC —including pregnant adolescents— will be invited to consent to the collection and inclusion of their health data in the trial analyses. In the intervention facilities, these women will also be invited to participate in group care.  Pregnant women will be followed until 42 days after birth. Pregnant women must be included as they are the target of this intervention, which has been shown to have positive effects in US populations. The outcomes of their neonates will be included as an additional outcome. This project has received local permission to waive parental consent for pregnant minors over the age of 15. They are included because they have been shown to especially benefit from this intervention. Rwanda's main languages are Kinyarwanda and French. Thus, providers and women may not be able to read, speak, or understand English.  The baseline and endline participant women's survey will measure locus of control, social support, pregnancy-related anxiety, and antenatal/postnatal knowledge. These same women will be asked at up to 12 weeks after birth to participate in a follow-up survey, 8-week Postnatal Participant Survey that will repeat the same set of questions asked at baseline about locus of control, social support, pregnancy- related anxiety, and antenatal/postnatal knowledge. This postnatal questionnaire will also ask each woman to report whether or not she is currently using a contraceptive method and, if so, which method. This questionnaire has been tailored for women receiving standard ANC/PNC care and women receiving gANC  /PNC. These surveys will aid us in assessing the effect of group and standard care on the above mentioned indicators. These findings may contribute additional evidence about the positive effects of group antenatal care compared to standard care.  The purpose of *baseline and follow-up provider surveys* is to capture changes in provider job satisfaction, motivation, and perceptions over time and analyze associations. Standard care providers will be asked questions about job satisfaction as were asked at baseline.  Group care providers are also asked these same questions about job satisfaction, with additional queries about whether they prefer to deliver group ANC or PNC versus standard ANC or PNC. As this questionnaire will be administered longitudinally, any inter- or intra-provider differences in job satisfaction, will be reported. Findings from these surveys may support other findings in the literature about the positive relationship between group care and provider job satisfaction.  Qualitative work with women and providers (including community health workers and other health worker staff involved in the group care process) will be conducted between 9 and 18 months after implementation to help inform the improvement of the model.  Master Trainers will observe providers in all intervention health centers to ascertain group care model fidelity.  We will collect data at delivery regarding the newborn (e.g. weight, gestational age at birth, sex, length, head circumference, chest circumference, mid-upper arm circumference and foot length), but this information will be gathered from the mother’s medical chart and the maternity ward register. For other outcomes related to the infant that extend past the time of delivery (e.g. 28-day and 42-day mortality |  |

| status), we will again obtain this information from data that is linked to the mother’s medical record. The primary data sources will be the neonatal and post-natal registers which allow us to link the infant to the mother’s record. Thus, while data regarding newborn measures and postnatal status will be collected, we will not interact or intervene with neonates directly, and these data are being collected as outcomes of the mothers’ pregnancy. Of note, all data are currently collected as routine care and will also be anonymized upon aggregation.  In order to ensure that the mother understands this component of data collection, we have already included this information in consent forms 4a-4f under the Procedures section. Specifically, these forms state, “….If you agree to allow us to collect your data, our study team will collect your reproductive and newborn data throughout your pregnancy and postpartum. This means they will review your file after each visit and record information about the type of care you received and your health. They will follow you throughout your pregnancy and your delivery even if you deliver at the district hospital.” Additionally, the study states that “we will follow your pregnancy from now until 42 days after you give birth.”  Describe the additional safeguards that have been included in the study to protect the rights and welfare of these subjects and minimize coercion or undue influence:  Here are some examples:  evaluating capacity to consent for individuals who may be decisionally impaired (specify how)  calibrating payment amounts to be non-coercive for the financially disadvantaged  conducting more in-depth evaluations of subjects’ understanding of the study and the voluntary nature of participation  involving advocates in the consent process  More information and other safeguards are described here: [**Vulnerable Subject**](http://irb.ucsf.edu/node/896) [**Populations**](http://irb.ucsf.edu/node/896) and [**Recruiting Staff and Students**](http://irb.ucsf.edu/recruitment#special).  The research team will be trained in ethical practices in human research. Special attention will be given to the process of obtaining informed consent without coercion for all aspects of the proposed study.  Research staff will emphasize that participation in the study is voluntary and that refusal to participate in the study will have no repercussion whatsoever.  The study will need to obtain informed consent from all participants, including women and providers.  Providers in the group care facilities will be asked to consent to observation of their fiedlity to the group care model, participation in surveys, and focus group discussions. Providers in the standard care facilities will be asked to complete surveys and focus group discussions. Again, special attention will be given to the process of obtaining informed consent without coercion. Research staff will emphasize that participation in the study is voluntary and that refusal to participate in the study will have no repercussion whatsoever.  Interviews and surveys with women and providers will be administered in private rooms at a time and location convenient to participants. Questionnaires will be administered, de-identified and protected by site coordinators to ensure anonymity and confidentiality. Focus group discussions will take place in private spaces. Names and other identifying characteristics will not be recorded on written or audio- recorded files. All files will be stored on password protected tablets and computers, and only project staff will have access to the files. Note that all audio files will be deleted after transcription and no identifying information will appear on transcribed files. Electronic documents will be shared with UCSF weekly using encrypted emails. |  |
| --- | --- |

| Any unique identifiers, such as names, and medical ID number, will be excluded from all data sets and kept in a separate secure dataset accessible to the local field team for tracking and linking purposes between different sources only. Each study participant will be assigned a study ID which will remain as the only case identifier in the analysis data set.  Prior to all data collection, the research team will administer consent to participants. Each participant will be given the opportunity to read the consent form or if illiterate have it read to her/him by a witness. The c onsent statement will explain the study objectives, requirements, potential risks, privacy and ethical obligations of the research team. The participant will complete the consent process by agreeing or disagreeing to the consent statement.  The consent forms will be administered in Kinyarwanda (the local language) and only participants who agree to participate and sign the consent form will participate.  Throughout the course of the trial, the study team will monitor and report any adverse events or protocol violations. Adverse events will be reported to RNEC and the UCSF IRB within 48 hours of discovery of said events and annual renewal reports will include summary tables of all adverse events and protocol violations. Protocol violation reports will be reviewed by the study team and as needed study staff or providers will receive remedial training to ensure compliance with protocol. Adverse events include maternal death, verbal or physical assault in group visit, or a breach of confidentiality by group participants. Although maternal deaths may well be unrelated to study participation, the event is of sufficient gravity that we will report all maternal deaths that occur in our study population. Protocol violations include lapses in adherence to study procedures such as improper enrolling or consenting, and deviation in the expected conduct of group care per study protocol. |  |
| --- | --- |
| **10.7 INCLUSION CRITERIA: Briefly describe the population(s) that will be involved in this study. Include anyone that data will be collected from or about (e.g. patients, healthy controls, caregivers, providers, administrators, students, parents, family members, etc.):** | |
| ***Site-level inclusion criteria***  *S*tudy sites that have at least 2 ANC providers were included in the final selection of 37 sites, of which 36  will be pair-matched and included.  ***Individual-level inclusion criteria-Group and Standard Care (Women)***  At both intervention and control facilities, all women who enroll in ANC —including pregnant  adolescents— will be invited to consent to the collection and inclusion of their health data in the trial analyses. In the intervention facilities, these women will also be invited to participate in group care.  The primary data analysis will be restricted to those women in the intervention and control facilities who present for ANC 1 before 24 weeks gestation and have attended more than 1 ANC visit at the health facility. But secondary analyses of all women presenting for ANC 1 at any gestation will also be completed.  Since the intervention will be “phased in” at health facilities randomized to group care, each matched pair of health facilities will begin enrolling women with a pregnancy due date on or after a certain day. This day is: *The group care start date (of the intervention site in the pair) + 18 weeks.* For example, if the group care start date is January 30, 2017, in both sites of the matched pair women with a pregnancy due date of June 5 or later will be enrolled in the trial.  ***Individual-level inclusion criteria-Group Care (Providers)***  *At our intervention sites, providers who offer maternal and child health related services will be invited to provide group care in their health center. All providers who are trained in group care will be sampled for baseline and follow up surveys, and will be consented for observations although whether they are observed will depend on scheduling.*  ***Individual-level inclusion criteria-Standard Care (Providers)*** |  |

| *At our control sites, providers who offer maternal and child health related services will continue to provide standard care. All providers who are consented will be sampled for baseline and follow up surveys*  Individual-level inclusion criteria-Group Care-Focus Groups (Women)  Women enrolled in group ANC/PNC will be invited to participate in focus group discussions. Individual-level inclusion criteria-Group Care-Focus Groups (Providers and CHWs)  Providers who offer group care will be invited to participate in focus group  discussions. Two facilities will be selected by the co-Principal Investigators and several staff from that site will be invited to participate in a focus group discussion related to their implementation success; this focus group may include providers, the head of health center, administrative staff, and CHWs associated with this single selected facility.  Individual-level inclusion criteria-Group & Standard Care-Surveys (Women)  Women enrolled in group and standard care will be invited to complete baseline, mid-point, and endline surveys.  Individual-level inclusion criteria-Group & Standard Care-Surveys (Providers and CHWs)  Providers who offer group and standard care will be invited to complete baseline, mid-point and endline surveys.  Individual-level inclusion criteria-Group Care-Observations (Providers and CHWs) Providers who offer group care will be observed during group care sessions. |  |
| --- | --- |
| **10.8 EXCLUSION CRITERIA: List any exclusion criteria (e.g. reasons why someone would not be included in the study):** | |
| ***Site-level exclusion criteria***  We excluded sites that have less than 2 ANC providers.  ***Individual-level exclusion criteria-Women***  Women who do not return to the health facility after ANC 1 for any further ANC will be excluded from the primary analysis. Those women who do not provide consent for data collection will be excluded from the trial.  Because the intervention will be “phased in” at health facilities randomized to group care, each matched pair of health facilities will begin enrolling women with a pregnancy due date on or after a certain day. This day is: *The group care start date (of the intervention site in the pair) + 18 weeks*. For example, if the group care start date is January 30, 2017, in both sites of the matched pair women with a pregnancy due date of June 5 or later will be enrolled in the trial. Women with a due date before June 5, 2017 will be excluded from the trial. These exclusion criteria apply to surveys and focus groups for women in group and standard care.  ***Individual-level exclusion criteria-Providers***  Providers who do not offer maternal and child health related services in health centers will be excluded from the study. |  |
| **10.9 * RESEARCH CONDUCTED ON PATIENT CARE WARDS: Do any study activities take place on any patient care units including inpatient wards, peri- or post-operative care units, operating rooms, or in the Emergency Department at UCSF Health medical facilities: (REQUIRED)** | |
| Yes No |  |

|  |  |
| --- | --- |
| **10.10 * INTENSIVE CARE NURSERY (ICN): Will you be enrolling any babies who are admitted to the Intensive Care Nursery (ICN) (this includes critically ill babies as well as lower acuity patients who need overnight monitoring and support): (REQUIRED)** | |
| Yes No |  |
| **10.11 * EMERGENCY DEPARTMENT: Does your protocol or study involve any of the following patient related activities in the emergency department (e.g. subject identification, recruitment, consent, blood draws, specimen retrieval, involvement of ED staff (nursing, tech, and/or physician), or any other ED based procedures): (REQUIRED)** | |
| Yes No |  |
| **11.0 Inclusion of Minors in Research** | |
| **11.1 REGULATORY CATEGORIES OF RESEARCH: Select all the** [**regulatory categories**](http://www.hhs.gov/ohrp/humansubjects/guidance/45cfr46.html#46.404) **that apply:** | |
| No greater than minimal risk (45 CFR 46.404, 21 CFR 50.51)  Greater than minimal risk but presenting prospect of direct benefit (45 CFR 46.405, 21 CFR 50.52)  Greater than minimal risk (though only a minor increase over minimal risk) and no prospect of direct benefit but likely to yield generalizable knowledge about the subjects disorder or condition (45 CFR 46.406, 21 CFR 50.53)  Research not otherwise approvable which presents an opportunity to understand, prevent, or alleviate a serious problem affecting the health or welfare of children (45 CFR 46.407, 21 CFR 50.54)  Explain why the research in this study falls under the above category or categories:  Pregnant adolescents will be offered to participate in group care and/or have their data collected and analyzed. They will be required to receive parental consent and to complete an assent form in order to participate in the study. The risks to them as participants in this study are minimal. During the group antenatal care visits they may decide to share information. Sensitive topics may be discussed but they are free to discuss or keep quiet. We will encourage all women in the groups to respect each other’s  privacy. If they decide that they do not want to allow us to use the information about their pregnancy and birth, they or their parents may withdraw their participation at any time.  Many adolescents enjoy this type of care and some studies have shown it can improve outcomes for some adolescents. Their participation will help us learn more about the impact of the implementation a group antenatal care model in Rwanda. We hope, in turn, that this information will help provide information to the government of Rwanda on how to improve antenatal and postnatal care services. |  |
| **11.2**  **MINORS CONSENTING: Will this study enroll minors who can** [**legally consent for themselves**](http://irb.ucsf.edu/node/826#exceptions) **(as in the case of emancipated minors or minors being treated for pregnancy or drug use without their parents knowing). This is different from agreeing to be in the study even when their parents are the ones providing 'official' consent, which we refer to as 'providing assent':**  **Note: This is very rare and the answer is usually 'No.'** | |
| Yes No  * Indicate why they can consent for themselves (check all that apply):  **(REQUIRED)**  The research is limited to conditions for which minors 12 years and older can legally give consent to treatment in the state where the research is being conducted. In the state of California, this includes |  |

| treatment for mental health or substance use disorders, the diagnosis and treatment of reportable communicable diseases, or care provided for sexual assault  The minors are seeking care related to the prevention or treatment of pregnancy  The requirement for parental consent may pose a risk to the personal safety or housing status of the minor  The minors are self-sufficient (e.g. homeless or living on their own) The minors are legally emancipated  Other (describe below)  **For studies involving drug and alcohol use, the minor’s parents or legal guardians may obtain medical information relating to the minor’s drug or alcohol abuse care even if the minor objects, unless the principal investigator obtains a** [**Certificate of Confidentiality**](http://irb.ucsf.edu/node/406). |  |
| --- | --- |
| **11.3**  **PARENTAL PERMISSION VS. WAIVER: Please review the** [**guidance**](http://irb.ucsf.edu/node/826) **to see under what circumstances the IRB can waive parental permission.** | |
| Parental permission will be obtained  Waiver of parental permission is requested: The waiver meets the provisions for a waiver of consent (i. e., the research poses minimal risk, it could not practicably be carried out without the waiver of parental permission, AND the waiver will not adversely affect the rights and welfare of the minor participants (45 CFR 46.116(d))  Waiver of parental permission is requested: Parental permission is not a reasonable requirement to protect the minor (e.g. neglected or abused children) or parental knowledge of the study may endanger the health or welfare of the minor (45 CRF 46.408(c))  Provide a brief justification for the waiver:  In Rwanda pregnant teens are allowed to consent to their own healthcare. A waiver has been obtained by the University of Rwanda to allow pregnant teens ages 15 and older to participate in this research study. We are trying to follow local ethical procedures and request the waiver. This research study poses minimal risk and does not adversely affect the rights of pregnant minors.  Provide details on the other protections that will be in place:  The same protections used for all women participating in this study will also be used for pregnant minors. No additional protections will be added. |  |
| **11.4 ASSENT OF MINORS OR WAIVER: Please review the** [**guidance**](http://irb.ucsf.edu/node/826#consent) **to see under what circumstances the IRB can waive assent.** | |
| Assent of children developmentally and psychologically able to provide assent will be obtained Waiver of assent is requested: The capability of some or all of the children is so limited that they  cannot reasonably be consulted  Waiver of assent is requested: The research holds out a prospect of direct benefit that is important to the health or well-being of the children and is available only in the context of the research  Waiver of assent is requested: The activities involving the minor are limited to chart review or the something equally innocuous  Waiver of assent is requested: It is not culturally appropriate to involve the minor in the decision to participate (e.g. some foreign research) |  |
| **11.5 DOCUMENTATION OF PERMISSION AND ASSENT: (select all that will be used):** | |
| Permission form addressed to the parents  Simplified assent form addressed to the child, 7-12 years old (parents get separate form) Assent form addressed to the child, 13 years and older (for subjects and parents)  Assent form addressed to the child, 13 years and older (parents get separate form) |  |

| Check one:  One parent's signature will be obtained Two parents' signatures will be obtained  If this study is approvable under regulatory category .405 and you plan to get permission from only one parent, explain why you think one parent's permission is sufficient:  Legally, pregnant minors are allowed to consent to their own antenatal and postnatal care. Therefore we are requesting a waiver of parental signature for pregnant adolescents ages 15 and older in section 11.3. This waiver has already been granted by the Rwandan National Ethics Committee. |  |
| --- | --- |
| **11.6 WARDS OF THE STATE: Might this study enroll wards of the state:** | |
| Yes No |  |
| **12.0 Recruitment and Consent** | |
| **12.1 * COMPETITIVE ENROLLMENT: Is this a competitive enrollment clinical trial? By competitive enrollment, we mean that sites who do not enroll participants early may not get to participate at all: (REQUIRED)** | |
| Yes No |  |
| **12.2 * SUBJECT IDENTIFICATION METHODS: What kinds of methods will be used to identify potential participants for recruitment (check all that apply): (REQUIRED)** | |
| Review of patients' conditions, history, test results, etc. (includes patients seen in clinic, scheduled for surgery, a procedure, imaging, or tests, or seen in the Emergency Department as well as searching through medical record data for possible cohort identification)  Already approved recruitment registry  Re-contact of participants from the investigators' previous studies  Referrals from colleagues (attach the 'Dear Colleague' letter or other recruitment materials you will provide to colleagues)  Referrals from the community / word of mouth  Advertisements (flyers, brochures, radio or t.v. ads, posting on clinical research sites or social media, presentation of the study at community events/media, etc.)  Online recruiting tool (describe below) CTSI Recruitment Services unit  Posting on UCSF Clinical Trials, ClinicalTrials.gov or other publicly available clinical trial website Other method (describe below)  * Provide details about the subject identification methods: **(REQUIRED)**  Pregnant women who report to a health center for their first antenatal care visit will be recuited into the study. All women reporting for antenal care will receive information about the study and be asked to consent by either the health provider or a study Field Coordinator who will be assigned to that health center for the duration of the study.  Through urine pregnancy testing at the community level, CHWs may spread information about our study. |  |
| **12.4 DETERMINATION OF ELIGIBILITY: How, when, and by whom will eligibility for recruitment be determined:** | |
| At both intervention and control facilities, all women who present for ANC 1 —including pregnant adolescents— will be assessed for eligibility and invited by their health care provider to participate in the |  |

| trial. If they express interest, a field coordinator will consent the women in the intervention arm, while providers will consent in control arms. This consent allows for the collection and inclusion of their health data in the trial analyses, as well as completion of the enrollment form.  A portion of women in both arms will also be invited to fill out a Baseline Questionnaire. These same women will be asked at 0-12 weeks after birth to participate in a follow-up survey, 8-week Postnatal Participant Questionnaire. Eligibility will be determined by the research team and field coordinators will manage the consent process. Because we only require a subset of women to participate in this, Field Coordinators will be instructed to invite the first 5 women they have contact with each month. The postnatal questionnaire will be administered in person by the data collector at the health facility. If we are unable to identify sufficient numbers of matched women at postnatal, we will recruit enrolled women for post-natal care questionnaire.  The baseline provider questionnaire will be administered to a cohort of providers across all study sites at the initiation of the trial—the 3-4 providers at each site who most regularly provide maternal health services. When additional providers are trained in group ANC/PNC, they will complete a baseline questionnaire at the time of training. The Follow-up Provider Questionnaire will be administered to this longitudinal sample of providers ideally 9 months and 18 months after they completed the baseline questionnaire, with questions tailored specifically to standard care and group care providers. Providers will be considered eligible if they provide antenatal or postnatal care services in a control facility, or in an intervention facility, if they received training to provide group antenatal and postnatal care services.  Qualitative work with women enrolled in group care, community health workers, providers, and some administrative health center staff will be conducted between 9 and 18 months after implementation to inform any fine tuning to the model and its provision. Women will be screened by the research team to ensure that they enrolled in group care during pregnancy.  At the time of group ANC/PNC training, the research team will consent group care providers for observations by Master Trainers. |  |
| --- | --- |
| **12.5 * INITIATION OF CONTACT: Who initiates contact (check all that apply): (REQUIRED)** | |
| Investigators/study team  UCSF recruitment unit (e.g. CTSI Consultation Services) Potential participant  Other (explain below)  Provide details about how contact is initiated:  For women, contact is initiated by their ANC provider during their first ANC visit or by a Community Health Worker who administers a urine pregnancy test and refers women to their nearest health center for an initial ANC visit, if pregnant. |  |
| **12.6 * HOW IS CONTACT INITIATED: (check all that apply): (REQUIRED)** | |
| In person Phone  Letter / email Website or app  Other (explain below) |  |
| **12.7 RECRUITMENT PLAN: Based on the checkboxes you chose above, please provide a narrative describing your recruitment plan. We want to know:**  **Who is conducting the search for potential participants, and how?**  **How are potential subjects being approached for recruitment? By whom, and when?**  **If there will be more than one participant group (e.g. patients, healthy controls, caregivers, family members, providers, etc.), provide details about the recruitment plans for each group. (Recommended length - 100-250 words)** | |
|  |  |

| At both intervention and control facilities, all women who present for ANC 1 —including pregnant adolescents age 13 and up—— will be assessed for eligibility and invited by their health care provider to participate in the trial. If they express interest, a field coordinator will consent the women. This consent allows for the collection and inclusion of their health data in the trial analyses, as well as completion of the enrollment form.  The primary data analysis will be restricted to those women in the intervention and control facilities who present for ANC 1 before 24 weeks gestation and have attended more than 1 ANC visit at the health facility. But secondary analyses of all women presenting for ANC 1 at any gestation will also be completed.  At intervention and control facilities, women in all arms of the study will be invited by the research team to complete baseline and endline surveys. Enrolled women will be asked at 4-12 weeks after birth to participate in a follow-up survey, 8-week Postnatal Participant Questionnaire. The Field Coordinator will ask the women waiting for immunization if they a) are enrolled in the Preterm Birth Initiative (PTBi) study cohort and b) completed the Baseline Participant Questionnaire. Any woman who is both enrolled in the PTBi study cohort AND completed the Baseline Participant Questionnaire is prioritized for participation in the Postnatal Participant Questionnaire, before the newborn receives any immunizations. (Based on experiences in the study thus far, we do not expect the Field Coordinators will locate many women who participated in the Baseline Participant Questionnaire.) These women will have already signed a consent form that includes administration of the Postnatal Participant Questionnaire. Next, the Field Coordinator will count the number of women present for immunization services who are enrolled in the PTBi study cohort, and divide this number by 5. The result of this calculation will produce a sampling interval to be used that day to select the women who will be invited to participate in the Follow-up Questionnaire.  The baseline provider questionnaire will be administered to providers across all study sites at the initiation of the trial. The Follow-up Provider Questionnaire will be administered to the same sample of providers 9 months and 18 months after the baseline questionnaire is completed. Recruitment will occur in health centers by the research team.  Qualitative work with women enrolled in group care, providers, and CHWs will occur between 9 and 18 months after implementation. The research team will recruit providers and CHWs associated with study sites to participate in the focus group discussions. Women enrolled in the study who are at least 6 weeks postpartum will be recruited for focus group discussions by the research team.  Those providers and CHWs trained in group ANC/PNC will be invited to consent to be observed during site visits by Master Trainers. |  |
| --- | --- |
| **12.8 * CONSENT METHODS: How will permission to participate (i.e., informed consent) be obtained from each potential participant. If there will be multiple groups and different plans for consenting each, check all that apply. See the orange Help bubble to the right for more detailed guidance. Participants will (check all that apply): (REQUIRED)** | |
| Sign a consent form at the end of the consent discussion (signed consent) Provide online 'eConsent' using an E-Signature system  Click through a link in a survey or email after reading about the study and then complete the study online (electronic consent)  Be told about the study and be given a handout/information sheet and be asked if they agree to participate (verbal consent)  Complete the study activities and turn in materials, as in the case of a completed survey that is placed in a drop box or mailed to the study team (implied consent)  Not be able to provide consent and will have a family member consent for them, as in the case of a critically ill or unconscious patient (surrogate consent)  Not be able to provide consent (emergency waiver of consent - allowed for minimal risk research or greater than minimal risk research with an approved community consultation plan)  Not know about the study, as in the case of chart reviews or observations of public behavior (waiver of consent)  Other method (describe below)  **Attach your consent form, information sheet, or electronic consent text in the Informed Consent Documents section of the Initial Review Submission Packet Form.** |  |
| **12.9 * CONSENT PROCESS: Describe the process for obtaining informed consent, including details such as who will have the consent discussion and when participants will be asked to sign the consent form in** | |

| **relation to finding out about the study: (REQUIRED) We encourage researchers to review our** [**guidance on obtaining and documenting informed consent**](http://irb.ucsf.edu/obtaining-and-documenting-informed-consent)**.**  **If there are multiple groups being consented differently, provide details about the consent process for each group.**  **If you are relying on** [**verbal or implied consent**](http://irb.ucsf.edu/node/292)**, provide details about how that will happen. For studies using online recruitment and consent or consent via mail, provide details here.** | |
| --- | --- |
| At enrollment into standard ANC at a health center, field coordinators will approach women, introduce themselves, assess eligibility, and begin the consent process. The consent statement will explain the study objectives, requirements, potential risks, privacy and ethical obligations of the research team. This will be followed by a second stage consent process which requires participants to demonstrate their understanding of the nature and requirements of the research including understanding that participation is voluntary and that information is kept private. Participants who are unable to provide consent or who are unwell during this first visit may be re- offered to enroll in group care at their next visit, if they still meet the eligibility criteria. Since the national Rwandan Ethics Committee has granted a waiver for adolescents ages 15 and older, they will now be enrolled in the trial following the same procedure as that for adult women.  For pregnant adolescents ages 13 and 14, special considerations will be necessary for adolescents. Specifically, field coordinators will explain the study objectives, requirements, potential risks, privacy and ethical obligations of the research team. This will be followed by a second stage consent process which requires parents to demonstrate their understanding of the nature and requirements of the research including understanding that participation is voluntary and that information is kept private.  At control sites, participants were initially enrolled by the health facility personnel, but will now be enrolled by study personnel as we have expanded our team. They are consenting only to the ongoing collection of data and nothing in their care is changing. Enrollment processes will include entering participant contact detail, medical history in the enrollment form and completion of the consent process by agreeing or disagreeing to the consent statement read by the participant herself or by a witness if the participant is illiterate. The consent statement will explain the study objectives, requirements, potential risks, privacy and ethical obligations of the research team. This will be followed by a second stage consent process which requires participants to demonstrate their understanding of the nature and requirements of the research including understanding that participation is voluntary and that information is kept private. Participants who are unable to provide consent or who are unwell during this first visit may be consented on a subsequent visit.  In order to obtain more responses for the Postnatal Participant Survey, a convenience sub-sample of women will be invited to participate in the Participant Survey at the time they present for postnatal care or newborn immunizations at study sites. Some of these women will not have consented to and participated in the Baseline Participant Survey. They will be invited to participate in the Postnatal Participant Survey. For baseline surveys among women and providers, the research team will administer consent at enrollment. According to the postnatal sampling interval described in Section 12.6, the Field Coordinator will approach a woman and explain the purpose and procedure of the Postnatal Participant Survey. The Field Coordinator will complete a full informed consent process through which each woman is able to voluntarily participate in the questionnaire or decline to participate. If written consent is obtained, the Field Coordinator will invite the woman to a private area where their conversation cannot be overheard. The Field Coordinator will then find the woman’s unique study ID in the tracking tool. This unique study ID will be recorded in the Postnatal Participant Questionnaire as it is administered by the Field Coordinator.  The research team will also administer consent for focus group discussions among providers and CHWs between 9 and 18 months after implementation.  * It is important that the people obtaining consent are qualified to do so. Briefly describe the training and experience these individuals have in obtaining informed consent: **(REQUIRED)**  As mentioned above, field coordinators will be hired and rigorously trained for a week in data collection and management; group care; consenting; and group care scheduling. They will receive training from our University of Rwanda and Rwanda Biomedical Center counterparts, with  support from the UCSF Monitoring, Learning, and Evaluation team. All health providers will also be trained on what study participation entails, so that they are able to ask questions if asked by their patients. |  |

| **12.10 * CONSENT COMPREHENSION: Indicate how the study team will assess and enhance the subjects' understanding of study procedures, risks, and benefits prior to signing the consent form (check all that apply): (REQUIRED) Tip: Review the Consent Comprehension - Learning Notes in the Help bubble at the right for specific questions that can be asked to assess comprehension, consider using the** [**UCSF Decision-Making Capacity Assesment Tool**](http://irb.ucsf.edu/sites/hrpp.ucsf.edu/files/decision-making-capacity-assessment-tool.docx)**, and review our** [**guidance on obtaining written or verbal informed consent**](http://irb.ucsf.edu/obtaining-and-documenting-informed-consent#obtaining) **for more detail on how to conduct the assessment.** | |
| --- | --- |
| The study team will engage the potential participant in a dialogue, using open-ended questions about the nature of the study or the experimental treatment, the risks and benefits of participating, and the voluntary nature of participation  Potential participants will be asked or shown a series of questions to assess their understanding of the study purpose, procedures, risks and benefits, as well as the voluntary nature of participation (especially appropriate when the consent process happens online or through a mobile health app)  Other method (describe below):  Provide details of the other approaches that will be used, if using another method to assess comprehension: |  |
| **12.11 * DECEPTION: Does this study rely on some deception or misinformation about what the researchers are observing to get valid data? (REQUIRED)** | |
| Yes No |  |
| **12.12 * NON-ENGLISH CONSENT METHOD: Indicate which** [**method(s)**](http://irb.ucsf.edu/node/371/) **you will use to consent non-English speaking subjects: (REQUIRED)** | |
| Preferred Method—Consent form and other study documents will be available in the subject’s primary language Personnel able to discuss participation in the patient’s language will be present for the consent process.  Short-Form—A qualified interpreter will translate the consent form verbally, and subjects will be given the Experimental Subject’s Bill of Rights in their primary language, following instructions in Those Who do not Read, Speak or Understand English for required witnessing and signatures  * Explain how you will maintain the ability to communicate with non-English speakers throughout their participation in the study: **(REQUIRED)**  All consent forms will be translated into the local language (Kinyarwanda). In addition, the research team will administer the consent process in the local language. |  |
| **12.14 TIME: What is the estimated time commitment for participants (per visit and in total):** | |
| What is the estimated time commitment for participants (per visit and in total):  We will collect data on all women (intervention and control) from ANC visit 1 until 42 days after they give birth. In intervention facilities, women will be invited to attend 4 total antenatal care visits and 1 postnatal care visit (1.5- to 2-hour commitment per visit). Women who complete questionnaires will be expected to commit approximately 30 minutes per questionnaire. We will also select a sub-sample of women in intervention facilities to complete focus group interviews (two hour time commitment). In total, each woman is expected to participate in the study throughout their pregnancy and 42 days after they give birth, although direct data collection includes only consent and enrollment(30 min) and surveys (30 min x 2 for a subsample) and focus groups (2 hours for a subsample). The care received as part of the group care session is expected to take about two hours per visit. While this is longer than a traditional one-on one visit it is richer contact and will be held at a pre-specified time unlike individual visits which are first come first serve and frequently require long wait times.  Providers in intervention facilities will be required to provide group care on all days their facilities assign them to antenatal care. At the conclusion of each group they will fill out a debrief form which will take 5-10 minutes. Observation of providers' facilitation of group care will occur during group care sessions. Provider questionnaires may require up to a 30 minute time commitment per survey. Providers and CHWs who participate in focus group discussions will be expected to commit approximately 2 hours of their time. |  |

| **IMPORTANT TIP: Ensure this information is consistent with the information provided in the consent form.** |  |
| --- | --- |
| **12.17 OTHER ALTERNATIVES: Describe other alternatives to study participation, if any, that are available to prospective subjects:** | |
| Women and providers have the option of opting out of group care. They may receive or provide standard antenatal care. Women also have the option of not attending antenatal care. |  |
| **13.0 Risks and Benefits** | |
| **13.1 RESEARCH-RELATED RISKS: Check if your study involves any of these specific research-related risks to participants that may need to be disclosed in the consent form:** | |
| Physical discomforts or pain  Risks to employment, or social or legal standing  Risk that the study team may observe possible evidence of child abuse, elder abuse, or a threat to self or others that they are required to report |  |
| **13.2 * RISKS: Describe any anticipated risks and discomforts not listed above: (REQUIRED)** | |
| First, breach of confidentiality can occur in the group care setting, whereby participants and/or providers, disclose personal information that may make an individual feel uncomfortable. Confidentiality breaches can lead to a variety of outcomes related to stigmatization, changes in employment and/or relationship  status. Second, the Participant Questionnaires include the use of the Edinburgh Postnatal Depression Scale (EPDS) at both baseline and 8-weeks postnatal. A score of 9 or higher, or a positive answer to the last question, is considered a positive screen for depression. |  |
| **13.3**  **MINIMIZING RISKS: Describe the steps you have taken to minimize the risks/discomforts to subjects. Examples include:**  **designing the study to make use of procedures involving less risk when appropriate minimizing study procedures by taking advantage of clinical procedures conducted on the study participants**  **mitigating risks by planning special monitoring or conducting supportive interventions for the study**  **having a plan for evaluation and possible referral of subjects who report suicidal ideation** | |
| The risks to participants in this study are minimal. During the group ANC visits they may decide to share information. Sensitive topics may be discussed but they are free to discuss or keep quiet. We will encourage group facilitators to look for verbal or nonverbal cues  that suggest that a woman is experiencing personal discomfort during group care sessions. During the consent process, we will also  encourage women to inform group facilitators of any personal discomfort during group care sessions. Women can refuse to participate  in a session or topic that makes them uncomfortable or withdraw participation at any time.We will encourage all women in the groups to respect each other's privacy.  The risk of breach of confidentiality will be addressed by establishing group norms in all groups and agreeing on the principle of not mentioning anything shared within the group. We expect this will be sufficient as all women will be sharing their views and by respecting others confidentiality can have theirs respected as well. These norms will apply to providers who act as facilitators as well, and as providers are accustomed to having personal information disclosed during medical visits, respecting confidentiality of the group is an extension of their usual professional ethics.  If women decide that they do not want to allow us to use information about their pregnancy and birth, they may withdraw  their participation at any time. The study team will ensure that field coordinators also emphasize to women that all of their information will be de-identified during analyses. |  |

| Similarly providers information will be de-identified and will not be presented at a level that allows identification. For example if a facility only has one provider then facility level will not be provided, but rather only district level, or trial-arm level data will be presented.  In addition, the Participant Questionnaires include the use of the Edinburgh Postnatal Depression Scale (EPDS) at both baseline and 8- weeks postnatal. A score of 9 or higher, or a positive answer to the last question, is considered a positive screen for depression.  Knowledge of a positive score may make a patient feel negative feelings, such as embarrassment or guilt, in addition to the current burden of depression. To minimize this risk, all field coordinators who administer these questionnaires will be trained to initiate referral for positive scores to the mental health/social work provider at the health center by giving the woman the information for how to access these resources. The field coordinators will also give the names of the women to the health center mental health/social work provider, but will not reveal the score, or the nature of the problem, only that the individual is in need of additional services. Women will be told that their name will be given to the mental health/social work provider at the health center, but that no specific information will be revealed. The mental health /social work providers may provide counseling or referral as per the system guidelines. |  |
| --- | --- |
| **13.5 * BENEFITS: (REQUIRED) Note: These are the benefits that the IRB will consider during their review. They are not necessarily appropriate to include in the consent form.** | |
| Possible immediate and/or direct benefits to participants and society at large (check all that apply):  Positive health outcome (e.g. improvement of condition, relief of pain, increased mobility, etc.) Closer follow-up than standard care may lead to improved outcomes or patient engagement  Health and lifestyle changes may occur as a result of participation Knowledge may be gained about their health and health conditions  Feeling of contribution to knowledge in the health or social sciences field  The research presents a reasonable opportunity to further the understanding, prevention, or alleviation of a serious problem affecting the health or welfare of children  Other benefit (describe below) None |  |
| **13.6 RISK TO BENEFIT RATIO: Explain why the risks to subjects are reasonable in relation to anticipated benefits, if any, to the participant or society:** | |
| The risks to subjects in this proposal are minimal, and the potential benefits greatly outweigh these risks and have the potential to inform an under researched topic. For data collection, we have gone to great lengths to use routine data sources wherever possible and minimize subject contact, thus minimizing risk and discomfort. |  |
| **13.7 * DATA AND SAFETY MONITORING: Do you have a Data and Safety Monitoring Plan (DSMP) for this study (A DSMP is required for Greater than Minimal Risk research): (Click the Help link for guidance on risk determination) (REQUIRED)** | |
| Yes No  **This is not required for minimal risk research but the UCSF IRB strongly recommends one to ensure the data collected are adequate to meet the research aims:** |  |
| **14.0**  **Data and Safety Monitoring Plan** | |
| **14.1 * DATA AND SAFETY MONITORING PLAN (DSMP): (REQUIRED) Provide a summary of the DSMP:** | |
|  |  |

| **All greater than minimal risk studies are required to provide a plan. Lack of an adequate plan is one of the most common reasons why IRB approval is delayed.**  **Instructions:**  Describe the plan for monitoring data quality and participant safety. Key areas that should be included in the plan are:  An explanation of the plan to monitor data collection, study progress, and safety  A description of who will perform the monitoring and at what frequency (e.g., the PI only, a contract research organization, a Data and Safety Monitoring Board or Data Monitoring Committee, etc.)  The type of data and events that will be reviewed (e.g., adverse events, breaches of confidentiality, unanticipated problems involving risk to participants or others, unblinded efficacy data, etc.)  Procedures and timeline for communicating monitoring results to the UCSF IRB, the study sponsor, and other appropriate entities  As appropriate:  A plan for conducting and reporting interim analysis Clearly defined stopping rules  Clearly defined rules for withdrawing participants from study interventions  This document provides guidance on the implementation of such standards of health related data reporting, specifically focusing on the quality of the data collection and assessment processes as well as procedures for feedback of health related data and/or information.  This document outlines the study team roles and responsibilities for data quality assurance.  In addition, the study will be monitored by a Data Safety and Monitoring Board. Please see the description of the DSMB bellow for additional information on what they will review and when.  Adverse events will be reported to the Data Manager by Field Coordinators. The Data Manager will gather all information and report to the Rwanda and UCSF PIS, who will report to the respective IRBS as per requirements.  Acronyms and Abbreviations ANC Antenatal Care  CHW Community Health Worker  cRCT cluster Randomized Control Trial  DQA Data Quality Assessment  DSMB Data and Safety Monitoring Board  HMIS Rwanda Health Management Information System MOH Ministry of Health  PNC Postnatal Care  PTBi EA Preterm Birth Initiative East Africa RBC Rwanda Biomedical Center  SPH School of Public Health  UPT Urine Pregnancy Testing |  |
| --- | --- |

| UR University of Rwanda  US Ultrasound  **STANDARD OPERATING PROCEDURES FOR DATA COLLECTION**  **Purpose**  To enable field coordinators and care providers to collect consistent data  To determine responsibility of field coordinators, care providers and investigators To document lost to follow up in the RCT  To provide information in relation to REDCap, participants outcomes, adverse event and ethical violations  **General Principles**  .All persons involved in the collection of participants-related information must ensure that standard forms and registers recommended by the Ministry of Health are consistently used for recording of patient data.  Each enrolled participant have to consent before collection personal information and a unique study ID number be provided for future follow ups. The consenting process is conducted by the field coordinators.  For any transfer-OUT, the transferring service unit or facility must ensure that all relevant records accompany the participant and the registers well filled to document the why, what and where the transfer was made to.  The medical record must be maintained in the health unit (i.e. service delivery site) where services are delivered.  All persons involved in the collection, management and use of patient-related information must ensure that the uses of those data do not "compromise" the confidentiality of data.  Information regarding the cRCT objectives will be captured in RedCap by the field coordinator within a period not exceeding 48 hours after the register has been filled  Field coordinators must synchronize data with the server immediately after completion of data entry whenever internet connection allows.  The Data and Safety Monitoring Board (DSMB) was set. The DSMB should review cumulative study data to evaluate safety, study conduct, and scientific validity and integrity of the trial, and therefore advise the study investigators.  Where internal data quality errors or omissions are identified, they must be dealt with and corrected as soon as possible.  This standard operating procedure recommends training on data quality and supervision.  **Data Quality Assessment**  The Field coordinators together with the investigators and the health providers must:   1. Choose 10% of record IDs/Study IDs to be re-done 2. Check for any missing data items in all data sources and dataset 3. Check for any missing records in all data sources including and dataset 4. Check for any missing participant dossiers, medical registers or data sets 5. Check for inconsistencies or errors across the different data sources including use of appropriate data collection tools, accurate transcription of data from/to appropriate data sources 6. Check for the consistent and appropriate use of standard forms and registers in the HC 7. Check for timeliness in recording information 8. Check for the accuracy of the daily, weekly, monthly, quarterly and/or annual report(s) by recounting from source documents 9. Check on the filing and storage condition of patient dossiers, registers and health facility reports 10. Review the data entry process in RedCap 11. Follow up on any action agreed during previous data quality assessments 12. If any error within any data source and dataset has been identified, it must be immediately corrected as close to the point of entry as possible. No level of inaccuracy should be viewed as acceptable. 13. The error rate should be within ± 5% for data submissions from each service area. Greater than 5% error rate will trigger remedial training of the FC and could result in termination if not   improved |  |
| --- | --- |

| **STANDARD OPERATING PROCEDURES FOR DOCUMENTS STORAGE AND RETENTION**  **Purpose**  This SOP has been developed to improve the management of records and consent:  To ensure that health related records are retained and stored securely in an appropriate manner such that they are available for use as required.  To ensure that signed consent forms are stored securely in an appropriate manner as transferred at the school of Public Health for permanent storage  To ensure confidentiality of participants’ records To avoid loss of, or missing data and information  **General Principles**  1. All study related records must be accurate and appropriately kept at the health centers All staff with access to health related records must respect the confidentiality issues.  Consent forms will be temporary kept in a locked cabinet at the health center and transferred t0 the School of Public Health to be securely stored for a long period of time.  **STANDARD OPERATING PROCEDURES FOR DATA QUALITY ASSURANCE**  **Purpose**  To maintain high quality data. The PTBi EA expect that all collected data must be accurate, complete, up- to-date, and consistently corrected when needed.  Cross-check HC reported numbers to DHIS2 with collected numbers in REDCap  **General Principles**  A data strengthening activity was conducted at the beginning of the RCT to ensure that data manager understand the data quality need and strategies to ensure quality  Investigators are required to quarterly assess data quality. However, every two weeks, data are discussed and issues raised for timely follow up.  **ROLES AND RESPONSIBILITIES**  **Health Centre In-Charge/Titulaire**  The Health Centre In-Charge/Titulaire:  Ensures that nationally approved standard patient forms, registers and other tools used for medical recording, staff files are available at all times in the facility  Ensures that procedures for data transcription are established and followed by Health Center staff Assigns appropriate and trained providers to conduct group care, ultrasound exam (where applicable).  Ensures staff attend all relevant training organized by the PTBi team in collaboration with MOH, RBC and UR  **Clinical Staff**  Ensure that all data collection tools being used are up-to-date  Ensure the complete, accurate and timely recording of participant data into the appropriate data collection tools (registers and charts)  Apply standards and best practices for data collection as defined by MOH Coordinate the consenting and enrollment process with the field coordinator  Facilitate the group care process and participate in debriefing at the end of each group Ensure that all laboratory tests are performed and results recorded in appropriate forms  **2.3.4. Field coordinators**  Ensure participants provide their consent before the enrollment |  |
| --- | --- |

| Works with the Clinical Staff to ensure that the data collected is complete, accurate and up to date Works with the Clinical Staff to ensure that the schedules for next participants visits are planned and monitored  Enter information and synchronize with the server on regular basis  Ensures the timely, accurate and complete data entry of records in RedCap, as required Ensure tracking tools are completed and share with the data manager on regular basis Ensure that quality errors detected by the investigators are responded to and fixed Coordinate with the data manager in planning supervisions by investigators Update/edit fields in REDCap forms as identified  **Data Manager**  Works with the field coordinators to ensure that the data collected is complete, accurate and up to date Compile all the tracking tools from field coordinators and triangulate with the collected data in RedCap  Ensures the timely, accurate and complete data checks of records as required and provide regular feedback to field coordinators  Organize supervisions for data quality assessment and maintain communication with field coordinators  Works with the biostatistician to ensure data are regularly analyzed and verified and data shared and discussed during the biweekly conference calls.  Weekly update tracking tools per Health Facility Weekly merge updated tracking tools  Conduct supportive supervision site visits Every quarter Weekly identify missing values across REDCap forms Update/Edit fields in REDCap forms  Weekly check for data completeness in REDCap Monthly review of completed forms  **Research Assistant**  Support the Data manager to regularly overseeing all day-to day aspects of data collection and reporting from field coordinators  Search published knowledge and summarize them to be shared with the rest of the team  Works with the data manager and the biostatistician to regularly clean, analyze data to update the rest of the team on the data quality and progress of data collection  Works with the field coordinators to ensure that the data collected is complete, accurate and up to date  Support the data manager to compile all the tracking tools from field coordinators and triangulate with the collected data in RedCap  Support the data manager to ensures the timely, accurate and complete data checks of records as required and provide regular feedback to field coordinators  Organize supervisions for data quality assessment and maintain communication with field coordinators  Organize and conduct supportive supervision site visits Every quarter Support the data manager to weekly check for data completeness in REDCap Monthly review of completed forms  Provide continual review of data quality, identify gaps  Monitor UPT data and Ultrasound records for completeness and accuracy and to determine uptake rates  **Rwanda Investigators**  Make close follow up should follow the data collection process and check for quality and consistency of data. Any inconsistency or incompleteness should be reported to the data manager for coordination and follow up  Conduct data analysis and quality checks on regular basis and timely generate tables for DSMB  Conduct supportive supervision site visits Every quartereekly share query/ list of identified issues with record ID/Study ID to the data manager Weekly check for data completeness in REDCap  **UCSF MLE Team** |  |
| --- | --- |

| Make close follow up should follow the data collection process and check for quality and consistency of data. Any inconsistency or incompleteness should be reported to the data manager for coordination and follow up  Conduct data analysis and quality checks on regular basis  Weekly share query/ list of identified issues with record ID/Study ID to the data manager Weekly check for data completeness in REDCap  Provide advice on how improving quality of data |  |
| --- | --- |
| **14.2 * DATA AND SAFETY MONITORING BOARD (DSMB): (REQUIRED) Will a Data and Safety Monitoring Board (DSMB) be established:** | |
| Yes No |  |
| **14.3 DSMB DETAILS: Provide details about the DSMB, including meeting frequency, and the affiliations and qualifications of members: Attach the DSMB charter to the Other Study Documents section. If the DSMB has not yet been established, submit details and the charter to us as soon as they become available.** | |
| Although this is a minimal risk study, we agreed with our in-country partners that it would be advantageous to voluntarily form a DSMB. The primary goals were: 1) to have an independent body monitoring progress of the trial and sample accumulation; and 2) to engage regional experts early on to enhance visibility of the study. The DSMB was not formed in response to any increase in patient risk.  ***DSMB Description***  This DSMB will be coordinated by research staff associated with the Preterm Birth Initiative (PTBi) Rwanda, including both the University of Rwanda (UR) and University of California, San Francisco (UCSF).  This DSMB will be independent of UR, UCSF, The Bill and Melinda Gates Foundation, regulatory agencies, Rwanda National Ethics Committee (RNEC), Institutional Review Boards (IRB), and investigators. None of the DSMB members should be involved directly in the study.  This charter will be approved by its DSMB members as attested to by signature of the chairperson.  ***DSMB Membership***  Members will disclose conflicts of interest and will be cleared of significant conflicts of interest and potential conflicts of interest in accordance with provisions in this charter.  DSMB members will sign confidentiality agreements covering DSMB activities.  Composition of membership will include at least one statistician, one midwife, two obstetrician- gynecologists, and one pediatrician.  All DSMB members will be citizens of countries located within the geography designated as “East Africa.”  Qualifications of membership include: statistician must have experience in clinical trials, midwife and physicians must have appropriate clinical experience in the field of obstetrics/gynecology and/or pediatrics.  DSMB members will be remunerated a small amount in association with each of three scheduled meetings. The members of this DSMB are currently:  Simon Kasasa, PhD: Biostatistician, Senior Lecturer, Makerere University School of Public Health, Kampala, Uganda |  |

| Florence Mirembe, MD: Obstetrician-gynecologist, Senior Lecturer, Makerere University School of Public Health, Kampala, Uganda  Cyprien Baribwara, MD: Project Director at the Center for International Health, Education, and Biosecurity (CIHEB) at the Institute of Human Virology, University of Maryland School of Medicine and the Country Director of Maryland Global Initiative Corporation (MGIC) Rwanda, a PEPFAR-funded HIV program of the University of Maryland in Rwanda.  Stephen Rulisa, MD: Obstetrician-gynecologist, Professor of Obstetrics & Gynecology and Dean of the School of Medicine & Pharmacy, University of Rwanda, Kigali, Rwanda  Sebalda Leshabari, PhD: Midwife, Senior Lecturer, Department of Community Health Nursing, Muhimbili University of Health and Allied Sciences, Dar es Salaam, Tanzania  ***Reporting***  Data reviewed by the DSMB will be provided by Sabine Musange, Principal Investigator of PTBi Rwanda, or designee.  Issues and recommendations identified by the DSMB will be provided to Dr. Sabine Musange (Principal Investigator, PTBi Rwanda) or Dr. Dilys Walker (Principal Investigator, East Africa Preterm Birth Initiative) by the DSMB chairperson in accordance with this charter.  Details of closed session deliberations (e.g., minutes) will be considered privileged and not subject to disclosure except as required by law.  ***Introduction***  The DSMB will function in accordance with the principles of the following documents:  ICH GCP ‘Note for Guidance on Good Clinical Practice’ found at: [**http://www.emea.europa.eu/pdfs**](http://www.emea.europa.eu/pdfs/human/ich/013595en.pdf)  [**/human/ich/013595en.pdf**](http://www.emea.europa.eu/pdfs/human/ich/013595en.pdf)  FDA ‘Guidance for Clinical Trial Sponsors: On the Establishment and Operation of Clinical Trial Data Monitoring Committees’ found at: [**http://www.fda.gov/CBER/gdlns/clintrialdmc.pdf**](http://www.fda.gov/CBER/gdlns/clintrialdmc.pdf)  Roles and Responsibilities  ***DSMB Roles and Responsibilities***  Meet periodically (see DSMB Meetings) to review aggregate and individual subject data related to safety, data integrity and overall conduct of the trial.  Review specific interim analyses for efficacy (see Study Review Criteria/Stopping Rules and Guidelines). Provide recommendations to continue or terminate the trial depending upon these analyses.  Communicate other recommendations or concerns as appropriate. Operate according to the procedures described in this charter.  Follow conflict of interest guidelines as detailed below (see DSMB Membership). Comply with confidentiality procedures as described below (see Confidentiality).  Maintain documentation and records of all activities as described below (see DSMB Meetings, DSMB Reports).  In addition, the DSMB, in collaboration with the trial investigators and PTBi staff, will be responsible for the following duties:   1. Creating a meeting schedule and format for meetings 2. Determining the format for data presentation 3. Specifying who will have access to interim data and who may attend all or part of the DSMB meeting 4. Developing procedures for assessing conflicts of interest 5. Overseeing the method and timing of interim analysis 6. Organizing report preparation and distribution 7. Examining and re-assessing total sample size   ***The PTBi research staff (at both UR and UCSF)***  Assure the proper conduct of the study. |  |
| --- | --- |

Assure collection of accurate and timely data (monitoring and data management). Compile and report Adverse Events (AEs) and Protocol Violations (PVs) to the DSMB. Promptly report potential safety concern

(s) to the DSMB.

Communicate with regulatory authorities, IRBs, and investigators, in a manner that maintains integrity (e. g., blinding) of the data, as necessary. (This communication is not the responsibility of the DSMB.)

Establishment of a DSMB and organization of meetings.

Prepare summary reports of relevant data for the DSMB. (This may include analyses not otherwise outlined in this charter based upon findings.)

DSMB Membership

The DSMB will consist of 5 members, listed in Appendix 1. The DSMB members have been selected by the PTBi research staff in consultation with the investigators and external advisors. The Chair of the DSMB will be selected by the PI and co-investigators, based on prior DSMB experience, scientific qualifications, willingness to commit an adequate amount of time to reviewing the trial data, and communication skills.

As characteristic qualifications, all members will:

Work professionally and meet qualifications for their respective professions as midwife, specialty physicians, and/or statistician.

Comply with accepted practices of their respective professions.

Comply with the conflict of interest policies to ensure that members do not have serious scientific, financial, personal, or other conflicts of interest related to the conduct, outcome, or impact of the study according to the guidelines specified below (e.g., engaged in any simultaneously occurring competitive trials in any role that could pose a conflict of interest for this study). See conflict of interest statement below.

Be independent from the funder, IRB, RNEC, regulatory agencies, principal investigator, co- principal or sub-principal investigator, site investigator, site sub-investigator, advisory board membership, clinical care of the study subjects, or any other capacity related to trial operations.

Not be on the list of Notice of Initiation of Disqualification Proceedings and Opportunity to Explain (NIDPOE) ([**http://www.fda.gov/foi/nidpoe/default.html**](http://www.fda.gov/foi/nidpoe/default.html)) and/or debarred list of investigators ([**http:**](http://www.fda.gov/ora/compliance_ref/debar)

[//www.fda.gov/ora/compliance_ref/debar](http://www.fda.gov/ora/compliance_ref/debar)).

Although each DSMB member will be expected to serve for the duration of the trial, in the unlikely event that a member is unable to continue participation, the reason will be documented and a replacement will be selected by PTBi staff.

Conflict of Interest

The DSMB will follow conflict of interest guidelines referenced by Department of Health and Human Services, Financial Relationships and Interests in Research Involving Human Subjects: Guidance for Human Subject Protection found at: [**http://www.hhs.gov/ohrp/humansubjects/finreltn/fguid.pdf**](http://www.hhs.gov/ohrp/humansubjects/finreltn/fguid.pdf) using as reference the EMEA Procedure on the Handling of Conflicts of Interests for EMEA Scientific Committees Members and EMEA Expert, found at: [**http://www.emea.europa.eu/pdfs/general**](http://www.emea.europa.eu/pdfs/general/direct/conflicts/ProcedureHandlingofConflictsofInterests.pdf)

[**/direct/conflicts/ProcedureHandlingofConflictsofInterests.pdf**](http://www.emea.europa.eu/pdfs/general/direct/conflicts/ProcedureHandlingofConflictsofInterests.pdf). DSMB members will sign a confidentiality and conflict of interest statement in regard to this study which will be on file with the PTBi staff. Conflicts of interest and/or potential conflicts of interest will be reduced to the greatest extent that is consistent with assembling a highly competent DSMB. Any questions or concerns that arise regarding conflicts of interest will be addressed by the DSMB chairperson with input from other DSMB members and PTBi staff as necessary.

DSMB Meetings

Projected Schedule of Meetings

The initial orientation meeting of the DSMB will be held so that members can review the charter, form an understanding of the protocol and definitions being used, confirm the meeting schedule and establish meeting rules, and review the study modification and termination guidelines. At all subsequent meetings, the DSMB shall examine and discuss study data for the interval preceding that meeting, review all AEs and PVs that occurred during the interval preceding that meeting, and any other information that the board deems as necessary.

| ***Timeline*** | ***Planned Date*** | ***Data Review by*** | ***Type of Information*** |
| --- | --- | --- | --- |
| Orientation meeting | September 2017 | Entire DSMB | Orientation materials for study and DSMB membership, data shells. |
| 9 months into enrollment | Feb 2018 | Entire DSMB | Adverse events listing, protocol violations listing, enrollment summary |
| 12 months into data collection | Sept 2018 – postponed and moved to February 2019** | Entire DSMB | Adverse events listing, protocol violations listing, enrollment summary, facility information, summary data tables, randomization data, |
|  |  |  | sample size calculations and |
|  |  |  | data on enrollment progress. |
| 15 months into data collection | February 2019** | Entire DSMB | Same as above |

** We held our initial two DSMB meetings in September 2017 and February 2018 as planned. June

meeting was delayed because not yet at 50%. When reached 50%, we realized problem with data collection item so needed to clean and format data before sharing with DSMB and are now planning our next meeting with DSMB in February 2019. All the timeline changes have been communicated to DSMB and all concurred in writing with ending enrollment in December 2018.

Meeting Format

DSMB meetings will generally be conducted by teleconference as members are spread across several countries. Any DSMB member present in Rwanda at the time of a scheduled meeting is politely requested to appear in person. If a DSMB strongly prefers to attend the meeting in person, and this necessitates travel from another country, PTBi can reimburse the member for actual travel expenses associated with DSMB meeting attendance only.

All other communication will be by email and teleconference. Meetings will be coordinated by PTBi staff. A quorum, defined as a minimum of three DSMB members, will be required to hold a DSMB meeting.

Critical decisions of the DSMB should be made by unanimous vote. However, if this is not possible, majority vote will decide. A facilitator (e.g., statistician or other representative from the PTBi staff responsible for report preparation) will attend the DSMB meetings as a non-voting member in order to facilitate data presentation and follow-up reporting, if deemed necessary by the DSMB. The meetings can include both open and closed sessions.

Study Review Criteria/Stopping Rules and Guidelines

Guidance for the conduct of safety and effectiveness analyses, and guidelines / stopping rules will be established prior to the DSMB’s first evaluation of data.

Safety Analyses

This is a study of a novel delivery model for routine ANC and PNC. Adverse events are not expected to be common, but may conceivably include: verbal or physical violence among group members; breach of confidentiality among group members; maternal death; or relatively poorer content of care at intervention sites.

Adverse Events are reported by any PTBi or health center staff who learn of their occurrence. Each AE is reviewed by the PI immediately and is determined to be unrelated or related to the study intervention(s). All AEs which are determined to be unrelated to study intervention will be collected and presented at DSMB meetings. All AEs which are classified as related, probably related or possibly related will be sent to the chairperson of the DSMB as they occur. AEs will be presented for the first time at the second DSMB meeting.

Stopping Guidelines / Stopping Rules: Safety

The DSMB may recommend termination or modification of the study if any of the following predefined conditions are met: if safety concerns arise, or if the intervention is judged to be so effective that withholding it from the control health facilities is judged to be unethical. In addition, termination or

| modification may be recommended for any other perceived safety concern based on clinical judgment, including but not limited to a high incidence of AEs.  ***Effectiveness Analyses***  The primary effectiveness endpoint is increased GA at birth. Secondary outcomes (attendance and preterm neonatal mortality) will also be considered in the effectiveness analyses. The DSMB will monitor effectiveness outcomes to determine relative risk/benefit, futility, or for early termination due to overwhelming effectiveness. As defined in the study protocol, the DSMB will review the interim analyses of effectiveness measures after enrollment of approximately 50% and 75% of participants (based on approximately 6 and 9 months of data at 20+ enrollees per health center per month) in both study arms.  ***Consideration of External Data***  The DSMB may also consider data from other studies or external sources during its deliberations, if available, if these results might have an impact on the status of the patients and design of the current study.  DSMB Reports  ***Monitoring for Safety***  The primary charge of the DSMB is to monitor the study for patient safety. Formal DSMB safety reviews will occur as specified above (see Study Review Criteria/Stopping Rules and Guidelines).  ***Monitoring for Effectiveness***  The DSMB will monitor effectiveness outcomes to determine relative risk/benefit, futility, or for early termination due to overwhelming effectiveness. DSMB effectiveness reports will occur as specified above (see Study Review Criteria/Stopping Rules and Guidelines).  ***Monitoring for Study Conduct***  The DSMB will review data related to study conduct. Data to be reviewed and listed in the DSMB reports includes: enrollment rates over time and summary of protocol violations and adverse events.  ***Data Flow for Adverse Events***  The DSMB will carefully monitor adverse events periodically throughout the duration of the study. At each meeting, the DSMB will review all reported adverse events and PVs. The Field Coordinators, PTBi staff, or health center staff are expected to report all Adverse Events to the Rwanda Principal Investigator and Project Manager and depending on the type of event, the PI will then inform the UCSF PI and the DSMB members. These guidelines are outlined below and follow UCSF protocols found at: [**http://www. researc**](http://www.research.ucsf.edu/CHR/Guide/Adverse_Events_Guidelines.pdf) [**h.ucsf.edu/CHR/Guide/Adverse_Events_Guidelines.pdf**](http://www.research.ucsf.edu/CHR/Guide/Adverse_Events_Guidelines.pdf)  *Clinical Events Committee*  A Clinical Events Committee will not be utilized for this clinical study.  ***Preparation of PTBi Rwanda CRCT Reports to the DSMB***  The PTBi staff will distribute reports of blinded data to the DSMB for the second and third DSMB meetings. The reports will be delivered by email approximately 14 days prior to the date of each DSMB meeting.  In order to provide the maximum amount of information to the DSMB, the analyses will employ the most recent data (recognizing limitations thereof) available at the time of the analysis. Requests for additional data by the DSMB members will be made to the DSMB chairperson or his or her designee, who will be responsible for communicating the request to the PTBi Rwanda PI.  The reports to be received by the DSMB can involve “open” and “closed” section  s. The open section will be made widely available, including to all study investiga tors, who may choose to share the report with IRBs, donors, and other interested parties. This section will include data in aggregate form focusing on trial conduct issues such as overall accrual and drop-out rates; eligibility rates and reasons for ineligibility; baseline characteristics; and compliance with protoc ol procedures. The DSMB can decide to have a closed section. If so, a report on t he closed section is at the DSMB discretion. The closed section of the DSMB report will be made available only to DSMB members. |  |
| --- | --- |

| ***DSMB Communication of Findings and Recommendations***  Following each meeting and within 30 days of the meeting, the chairperson will send findings and recommendations of the DSMB in writing to the PIs and PTBi staff.  These findings and recommendations can result from both the open and closed sessions of the DSMB. If these findings include serious and potentially consequential recommendations that require immediate action, the chairperson will also promptly notify Dr. Musange and Dr. Walker by phone.  ***PTBi’s Response to DSMB Findings and Recommendations***  Drs. Musange and Walker will review and respond to the DSMB recommendations. The recommendations of the DSMB will not be legally binding but require professional consideration by the recipients. If the DSMB recommends continuation of the study without modification, no formal response will be required. However, if the recommendations request action, such as a recommendation for termination of the study or modification of the protocol, the DSMB will request that PTBi investigators provide a formal written response stating whether the recommendations will be followed and the plan for addressing the issues.  It is recognized that the PTBi may need to consult with regulatory agencies or other consultants before finalizing the response to the DSMB. Upon receipt, the DSMB will consider the PTBi response and will attempt to resolve relevant issues, resulting in a final decision. Appropriate caution will be necessary during this process to avoid compromising study integrity or the ability of PTBi to manage the study, should the study continue. PTBi staff will agree to disseminate the final decision to the appropriate regulatory agencies and IRBs within an appropriate time.  In the unlikely event of irreconcilable differences, especially regarding study termination or other substantial study modifications, the DSMB may decide to discontinue monitoring the current study and disband. This decision will be communicated to the study investigators and the funder.  Public disclosure of PTBi’s final decision or DSMB recommendations will be at the discretion the funders or their designee. The DSMB will not make any public announcements either as a group or individually.  **DSMB Closeout**  This study may be terminated under a variety of circumstances including, but not limited to, termination for overwhelming effectiveness (existing statistically significant results demonstrating the effectiveness of the intervention over the current protocol, prior to reaching the target sample size), futility (evidence that, given the rate of enrollment or the results presented, it is impossible that the trial will demonstrate a result even if continued to its projected sample size), or safety issues (data revealing that the intervention in some way threatens the safety of participants) per protocol or DSMB monitoring guidelines.  Responsibilities of the DSMB with regard to closeout will be to review the final study report to ensure study integrity. The DSMB may recommend continuing action items to PTBi investigators.  Confidentiality  All data provided to the DSMB and all deliberations of the DSMB will be privileged and confidential. The DSMB will agree to use this information to accomplish the responsibilities of the DSMB and will not use it for other purposes without written consent from the study investigators. No communication of the deliberations or recommendations of the DSMB, either written or oral, will occur except as required for the DSMB to fulfill its responsibilities. Individual DSMB members must not have direct communication regarding the study outside the DSMB (including, but not limited to the investigators, IRB, regulatory agencies, or others).  Amendments to the DSMB Charter  This DSMB charter can be amended as needed during the course of the study. Information to be included as amendments will be any modifications or supplements to the reports prepared for the DSMB, as well as amendments to other information addressed in this charter. All amendments will be documented with sequential version numbers and revision dates, and will be recorded in the minutes of the DSMB meetings. Each revision will be reviewed and agreed upon by PTBi staff and the DSMB. All versions of the charter will be archived in accordance with this document (see Archiving of DSMB Activities and Related Documents).  Archiving of DSMB Activities and Related Documents  All DSMB documentation and records will be retained in two locations:  1. The East Africa PTBi office at UCSF Global Health Sciences, Mission Hall, 550 16th Street, 3rd floor, San Francisco, California, and |  |
| --- | --- |

| This DSMB’s documentation and records will be retained by the PTBi staff or individual responsible for archiving until discarded 3 years after completion of the study in accordance with Good Clinical Practice guidelines (‘Note for Guidance on Good Clinical Practice’ found at: [**http://www.emea.europa.eu/pdfs/h**](http://www.emea.europa.eu/pdfs/human/ich/013595en.pdf) [**uman/ich/013595en.pdf**](http://www.emea.europa.eu/pdfs/human/ich/013595en.pdf). |  |
| --- | --- |
| **15.0 Confidentiality, Privacy, and Data Security** | |
| **15.1 PROTECTING PRIVACY: Indicate how subject privacy will be protected:** | |
| Conduct conversations about the research in a private room  Ask the subject how they wish to be communicated with – what phone numbers can be called, can messages be left, can they receive mail about the study at home, etc.  Take special measures to ensure that data collected about sensitive issues do not get added to their medical records or shared with others without the subject’s permission  Other methods (describe below) |  |
| **15.2 SENSITIVE DATA: Do any of the instruments ask about illegal or stigmatized behavior:** | |
| Yes No |  |
| **15.3 SIGNIFICANT CONSEQUENCES OF A LOSS OF PRIVACY OR CONFIDENTIALITY: Could a breach of privacy or confidentiality result in any significant consequences to participants, such as criminal or civil liability, loss of state or federal benefits, or be damaging to the participant's financial standing, employability, or reputation:** | |
| Yes No  Check all that apply:  Embarrassment  Criminal or civil liability  Loss of state or federal benefits  Damaging to the participant's financial standing, employability, or reputation Potential risks to insurability (health, disability, or life insurance)  Describe the potential consequences:  During group care sessions, participants may share confidential information which may result in damage to their reputation in their communities. |  |
| **15.4 EXTRA CONFIDENTIALITY MEASURES: Explain any extra steps that will be taken to assure confidentiality and protect identifiable information from improper use and disclosure, if any:** | |
| We will firmly ask all participants during the consent process and group sessions to maintain group confidentiality in and out of group care. |  |
| **15.5 * REPORTABILITY: Do you anticipate that this study may collect information that State or Federal law requires to be reported to other officials, such as elder abuse, child abuse, or threat to self or others: (REQUIRED)** | |
| Yes No |  |
| **15.6 CERTIFICATE OF CONFIDENTIALITY: Will this study obain a Certificate of Confidentiality:** | |

| Yes No |  |
| --- | --- |
| **15.7 SHARING OF RESEARCH RESULTS: Will there be any sharing of EXPERIMENTAL research test results with subjects or their care providers:** | |
| Yes No |  |
| **15.8 * HIPAA APPLICABILITY - STUDY LOCALE: Will the study population reside EXCLUSIVELY in the foreign country (excluding U.S. military bases, which should be considered U.S. soil): (REQUIRED)** | |
| Yes No  **Why do we ask this? This question is used to determine applicability of HIPAA regulations. If there are also populations within the U.S. or involving U.S. citizens you should check 'No.'** |  |
| **15.9 * HIPAA APPLICABILITY: Study data will be: (REQUIRED)** | |
| Derived from a medical record (e.g. APeX, OnCore, etc. Identify source below) Added to the hospital or clinical medical record  Created or collected as part of health care Used to make health care decisions  Obtained from the subject, including interviews, questionnaires Obtained ONLY from a foreign country or countries  Obtained ONLY from records open to the public Obtained from existing research records  None of the above  Derived from the Integrated Data Repository (IDR) or The Health Record Data Service (THREDS) at SFGH  If derived from a medical record, identify source:  Antenatal card, maternity chart |  |
| **15.10 * IDENTIFIERS: Check all identifiers that will be collected and included in the research records, even temporarily: (REQUIRED)** | |
| Names Dates  Postal addresses Phone numbers Fax numbers  Email addresses  Social Security Numbers* Medical record numbers Health plan numbers  Account numbers  License or certificate numbers Vehicle ID numbers  Device identifiers or serial numbers Web URLs  IP address numbers Biometric identifiers |  |

| Facial photos or other identifiable images Any other unique identifier  None  * Could study records include *ANY* photos or images (even 'unidentifiable' ones):  **(REQUIRED)**  Yes No |  |
| --- | --- |
| **15.11 * PATIENT RECORDS: Will health information or other clinical data be accessed from UCSF Health, Benioff Children's Hospital Oakland, or Zuckerberg San Francisco General (ZSFG): (REQUIRED)** | |
| Yes No |  |
| **15.18 * DATA COLLECTION AND STORAGE: (check all that apply): (REQUIRED)** | |
| Collection methods:  Electronic case report form systems (eCRFs), such as OnCore or sponsor-provided clinical trial management portal  UCSF ITS approved Web-based online survey tools: Qualtrics or RedCap Other web-based online surveys or computer-assisted interview tool  Mobile applications (mobile or tablet-based) Text Messaging  Wearable devices  Audio/video recordings Photographs  Paper-based (surveys, logs, diaries, etc.) Other:   - What online survey or computer assisted interview tool will you use: **(REQUIRED)**   Qualtrics (Recommended) RedCAP (Recommended)  Survey Monkey (NOT recommended and may require UCSF ITS Security review) Other   - For each app and device, please provide: **(REQUIRED)**   the name of the mobile application or wearable device name of the manufacturer / application owner  the FDA status (required for mobile health applications and mobile health devices)   1. Sony voice recorder 2. Samsung galaxy 8 inch tablet   * Data will be collected/stored in systems owned by (check all that apply):  **(REQUIRED)**  Study sponsor  UCSF data center (including OnCore, RedCap, Qualtrics, and MyResearch)  UCSF encrypted server, workstation, or laptop residing outside of UCSF data center Personal devices, such as laptops or tablets that are not owned or managed by UCSF SF VAMC  Zuckerberg San Francisco General Hospital Benioff Children's Hospital Oakland |  |

| Langley Porter Psychiatric Institution  Other UCSF affiliate clinic or location (specify below)  Cloud vendor such as Amazon Web Services (AWS), Salesforce, etc. (specify below) Other academic institution  3rd party vendor (business entity) Other (explain below) |  |
| --- | --- |
| **15.20 * DATA SHARING: During the lifecycle of data collection, transmission, and storage, will identifiable information be shared with or be accessible to anyone outside of UCSF: (REQUIRED)** | |
| Yes No |  |
| **16.0 Financial Considerations** | |
| **16.1 * PAYMENT: Will subjects be paid for participation, reimbursed for time or expenses, or receive any other kind of compensation: (REQUIRED)** | |
| Yes No |  |
| **16.4 COSTS TO SUBJECTS: Will subjects or their insurance be charged for any study activities:** | |
| Yes No |  |
| **17.0 Other Approvals and Registrations** | |
| **17.4 OTHER APPROVALS: Indicate if this study involves other regulated materials and requires approval and/or authorization from the following regulatory committees:** | |
| Institutional Biological Safety Committee (IBC) Specify BUA #:  Institutional Animal Care and Use Committee (IACUC) Specify IACUC #:  Controlled Substances |  |
| **18.0 Qualifications of Key Study Personnel and Affiliated Personnel**  **NEW: January 2019 - Affiliated personnel who do not need access to iRIS no longer need to get a UCSF ID. Instead, add them below in the Affiliated Personnel table below.** | |
| **18.1 Qualifications of Key Study Personnel:** | |
| **Instructions:** |  |

For UCSF Key Study Personnel (KSP)* listed in **Section 3.0,** select the KSP from the drop down list and add a description of their study responsibilities, qualifications and training. In study responsibilities, identify every individual who will be involved in the consent process. Under qualifications, please include:

Academic Title

Institutional Affiliation (UCSF, SFGH, VAMC, etc.) Department

Certifications

**NOTE: This information is required and your application will be considered incomplete without it. If this study involves invasive or risky procedures, or procedures requiring special training or certification, please identify who will be conducting these procedures and provide details about their qualifications and training. Click the orange question mark for more information and examples.**

**Training Requirements:**

The IRB requires that all Key Study Personnel complete Human Subjects Protection Training through [**CITI**](http://www.citiprogram.org/) prior to approval of a new study, or a modification in which KSP are being added. More information on the CITI training requirement can be found on our [**website**](http://irb.ucsf.edu/citi-human-subjects-training)**.**

* **Definition of Key Study Personnel and CITI Training Requirements (Nov, 2015):** UCSF Key Study Personnel include the Principal Investigator, other investigators and research personnel who are directly involved in conducting research with study participants or who are directly involved in using study participants’ identifiable private information during the course of the research. Key Personnel also include faculty mentors/advisors who provide direct oversight to Postdoctoral Fellows, Residents and Clinical Fellows serving as PI on the IRB application.

| **KSP Name** | **Description of Study Responsibilities - Briefly describe what will each person be doing on the study. If there are procedures requiring special expertise or certification, identify who will be carrying these out. Also identify who will be obtaining informed consent.** | **Qualifications, Licensure, and Training** | |
| --- | --- | --- | --- |
| Walker, Dilys, MD | Principal Investigator - Dr. Walker will lead the East Africa-based research portfolio, including the implementation research and discovery research programs in Uganda, Kenya, and Rwanda. | Dilys Walker, MD, is an obstetrician gynecologist and a professor in the departments of Obstetrics, Gynecology, and Reproductive Sciences, and Global Health Sciences. Prior to joining UCSF, she worked at the University of Washington and was Associate Director for the Global Center for Adolescent and Women's' Health. She also worked for 11 years as an investigator in Reproductive Health at the National Institute of Public Health in Mexico. Dr. Walker and her team developed a novel approach to emergency training—PRONTO—using highly- realistic simulation and team training to improve obstetric and neonatal |  |

outcomes. She is President of the NGO PRONTO

International and is actively running PRONTO implementation trials in Mexico, Guatemala, and Kenya. Her team is currently working with CARE India to implement a mentor driven simulation and team-training program for Bihar, India.

Butrick, Elizabeth A Senior Program Manager - Elizabeth will lead efforts to implement our portfolio of East Africa research projects. She will provide coordination and oversight to the UCSF team and liaise with in country partners. She will monitor projects, maintain relationships with investigators, and support overall strategic planning for the PTBi East Africa including working with MLE, resource allocation, and reporting for the initiative.

Elizabeth Butrick has over 15 years experience managing research and implementation projects in maternal and child health in LMICs. For the last 9 years, her focus has been on maternal health projects in Sub- saharan Africa.

Santos, Nicole M Discovery Research Program Manager - Dr. Santos will primarily support our discovery research portfolio and the execution of capacity building efforts. Specifically, she will work with researchers to match their interests with PTBi platforms, and to provide technical assistance and coordination support to monitor progress. She will help develop relationships with stakeholders and ensure that discovery opportunities are integrated into implementation efforts wherever possible. As a core team member for PTBi EA, Dr. Santos is also providing support to execute the initiative’s implementation research aims.

Nicole Santos has 10 years experience in both basic science and global health research. Her previous work focused on cell signaling, infectious diseases, and diagnostics for low-resource settings. She has helped implement cohort studies in several LMICs, including the supervision of data/sample collection and laboratory staff training.

Myrick, Roger Director of Monitoring & Evaluation – Dr. Myrick will be responsible for oversight of project management and development and implementation of all PTBI MLE deliverables. He will provide high- level technical guidance to the Initiative, specifically in terms of cross- collaborative MLE efforts among each Aim group, and developing working relationships with the MLE team from the Bill and Melinda Gates Foundation. Dr. Myrick will liaise directly with the MLE

Roger Myrick is the Director of Monitoring and Evaluation (M&E) for the Prevention and Public Health Group (PPHG). He provides leadership for the M&E team and technical assistance in countries throughout sub-Saharan Africa, Southeast Asia and South America. Dr. Myrick has assisted with the development of Strategic Information (SI) and M&E capacity building guidelines for PEPFAR, and provided extensive SI support to PEPFAR technical working groups on M&E, prevention for

Officer of the PTBI Initiative at BMGF, and meet regularly with key personnel from the PTBI MLE group and the leadership team to ensure continuous feedback and seamless engagement and revision to all MLE activities.

general populations and youth, and prevention with positives. Dr. Myrick also coordinated the first multi- agency International M&E Field Officer program in 2004. Before joining PPHG in 2010, Dr. Myrick held various senior M&E advisory and management positions at UCSF as well as the U.S. Centers for Disease Control (CDC). His experience includes working for the CDC Global AIDS Program, the UC University-wide AIDS Research Program, and the California State Office of AIDS.

Azman Firdaus, Hana M Monitoring, Learning & Evaluation (MLE) Technical Advisor. Under the guidance of the Director of MLE, Hana will work closely with the PTBi RW and PTBI EA teams to ensure the data processes for this RCT — data collection, flow, and reporting — are of robust and sound quality.

Specifically, Hana will be communicating on a routine basis with the Data Manager of the study to provide technical support on routine data monitoring and periodic evaluation, along with systematic data strengthening efforts, due to the nature of the data sources (I.e., national tools) being used for this particular trial. Hana will also provide overall technical support for monitoring and evaluation of outcomes of the trial, as a deliverable to the Gates Foundation.

Hana Azman Firdaus is a Sr. Technical Advisor with multiple years experience working in international health settings. She has led the development of National M&E Plans in several countries, and was the lead M&E person for PEPFAR GBV Initiative in Tanzania, among others. Hana has extensive fieldwork experience ranging from facility assessments, supportive supervision site visits, and training of facility, district, and national health facility staff.

She has also been involved in the design and roll-out of both paper- and electronic based national data collection and reporting tools across multiple LMIC countries.

Lundeen, Tiffany B Tiffany Lundeen will serve as a group care curriculum developer and the point of contact for Rwandan stakeholders regarding provider training, facility planning, implementation and monitoring for model fidelity.

Tiffany Lundeen is a midwife with 12 years of experience delivering and teaching group care. She has additional training in human centered and health systems design.

Sterling, Mona A

Mona has a bachelor's degree.

Williams, Pamela G

Pamela is a masters candidate in Global Health.

Pamela is a student intern who will work on data analysis.

Mona Sterling is an admin assistant who will assist with IRB submissions.

Millar, Kathryn R

Kathryn is a masters candidate in Midwifery at the School of Nursing

Kathryn is a student intern who will work on data analysis.

Miller, Phoebe N Phoebe is a student intern who Phoebe Miller is a first year

will work on data analysis. Joint Medical Program student

interested in obstetrics- gynecology and prenatal health care. Before coming to medical school, she spent two years working in Johannesburg, South Africa. For a year she worked in an Emergency Department in Johannesburg, which was where she first became interested in the health disparities facing pregnant women. For her JMP Master's thesis she would like to continue studying interventions that support ante-natal care for women in under resourced settings.

Sparks, Aleah

Aleah Sparks is a graduate student researcher with the Preterm Birth

Initiative, East Africa. She worked with Preterm Birth Initiative, California

Aleah is a student who will work for this project as a research assistant.

as a Project Analyst and Research Coordinator since its launch in 2015.

Aleah has a bachelor’s degree in sociology and completed her RN pre-

licensure year at the UCSF School of Nursing. She will enter her advanced

practice training in September pending completion of her Board Exam. As

a Nurse Practitioner, Aleah’s specialty area will be in pediatrics with concentrations in genomics and global health. Her research interests

include long-term impact of prematurity on children and families and

quality of maternal-child healthcare in low resource settings.

Sloan, Nancy L

Dr. Sloan is an epidemiologist with over 30 years of experience in global maternal- child health studies and an author on 51 peer-reviewed journal articles.

Dr. Nancy Sloan will serve as the Senior Data Scientist for the East Africa Preterm Birth Initiative.

Phillips, Elizabeth S

Beth Phillips is an MPH with over 10 years experience in study coordination and data management and analysis in diverse and often remote settings across Africa and Asia.

Beth Phillips will serve as a project manager and will perform data analysis and cleaninig as needed. Her role may involve linking or checking data linking and thus includes access to personal identifiers

Schmidt, Christina N Christina, a current UCSF MD Aleah Sparks is a graduate

student, will be assisting with secondary data analysis on PTBi Rwanda datasets as part of her research program for her degree and assisting with other data cleaning checks and tasks as needed.

student researcher with the Preterm Birth Initiative, East Africa. She has degrees in anthropology and global health and has worked for several years on Maternal Health issues in Kenya before returning to school to pursue her medical degree.

Ghosh, Rakesh

Dr. Rakesh Ghosh will serve as the Data Scientist for the East Africa Preterm Birth Initiative specifically to oversee end of study dissemination efforts.

Dr. Ghosh has extensive experience leading study analyses, designs, and presentations for large global health trials.

**18.2 Affiliated Personnel:**

**Instructions:**

This section is for personnel who are not listed in **Section 3.0: Grant Key Personnel Access to the Study** because their names were not found in the User Directory when both the iRIS Database and MyAccess directories were searched. Add any study personnel who fit ALL of the following criteria in the table below:

They meet the definition of Key Study Personnel (see above), **and**

They are associated with a UCSF-affiliated institution (e.g., VAMC, Gladstone, Institute on Aging, Vitalant, NCIRE, SFDPH, or ZSFG), **and**

They do not have a UCSF ID, **and**

They do not need access to the study application and other study materials in iRIS.

**Note:** Attach a [**CIT**](http://www.citiprogram.org/)**I Certificate** for all persons listed below in the **Other Study Documents** section of the **Initial Review Submission Packet Form** after completing the **Study Application**.

Click the orange question mark icon to the right for more information on who to include and who not to include in this section.

Do not list personnel from outside sites/non-UCSF-affiliated institutions. Contacts for those sites (i.e. other institution, community-based site, foreign country, or Sovereign Native American nation) should be listed in the **Outside Sites** section of the application.

**If there are no personnel on your study that meet the above criteria, leave this section blank.**

| **Name** | **Institution** | **Telephone** | **E-mail** | **Role** |  |
| --- | --- | --- | --- | --- | --- |

No External Personnel has been added to this IRB Study

Please describe the study responsibilities and qualifications of each affiliated person listed above:

- 1. **End of Study Application**

|  | |
| --- | --- |
| **End of Study Application Form**  **To continue working on the Study Application**:  Click on the section you need to edit in the left-hand menu. Remember to save through the entire Study Application after making changes.  **If you are done working on the Study Application**:  **Important:** Before proceeding, please go back to Section 4.0 Initial Screening Questions and **Save and Continue** through the form to make sure all the relevant sections and questions have been included. If you've changed any answers since you started, the branching may have changed. Your application will be incomplete and it will have to be returned for corrections.  Once you are sure the form is complete, click **Save and Continue**. If this is a new study, you will automatically enter the **Initial Review Submission Packet Form**, where you can attach **consent forms** or other **study documents**. Review the [**Initial Review Submission Checklist**](http://irb.ucsf.edu/sites/hrpp.ucsf.edu/files/initial-submission-checklist.pdf) for a list of required attachments.  **Answer all questions and attach all required documents to speed up your approval.**  The UCSF IRB welcomes feedback about the IRB Study Application Form. Please click the link to answer a  [**survey**](https://ucsf.co1.qualtrics.com/SE/?SID=SV_b9KE0pEeNwrqUe1) about the application form. |  |

**NATIONAL ETHICS COMMITTEE/ COMITE NATIONAL D'ETHIQUE**

**Telephone:** (250) 2 55 10 78 **84 Ministry of Health**

### E-mail: [info@rnecrwanda.org](mailto:info@rnecrwanda.org) P.O. Box. 84

**Web site:** [www.rnecrwanda.org](http://www.rnecrwanda.org/) **Kigali, Rwanda.**

### FWAAssurance No. 00001973

**IRB 00001497 oflORG0001100**

February 20, 2017 No.0034/RNEC/2017

**Dr. Sabine Musange**

**Local Lead Investigator**

**UR**

Your Project title: **"Preterm Birth Initiative Group Care Randomized Controlled Trial"**

has been evaluated by the Rwanda National Ethics committee.

|  | | Involved in the decision | | |
| --- | --- | --- | --- | --- |
|  |  | Yes | No ( Reason) | |
| Name | Institute |  | Absent | Withdrawn from  the proceeding |
| Dr.Jean-Baptiste MAZARATI | Biomedical Services  (BIOSl | **X** |  |  |
| Pro f. Eugene RUTEMBESA | U.niv€rsity of Rwanda | **X** |  |  |
| Dr.Laetitia NYIRAZINYOYE | University of Rwanda |  | **X** |  |
| Mrs. Frarn;:oise UWINGABIYE | Lawyer at RUSIZI | X |  |  |
| Dr. Egide KAYITARE | University of Rwanda | X |  |  |
| Sr.Domitilla  MUKANTABANA | Kabgayi Nursing and  Midwife school | **X** |  |  |
| Dr. David K. TUMUSIIME | University of Rwanda | **X** |  |  |
| Dr. Lisine TUYISENGE | Kigali Teaching Hospital | **X** |  |  |
| Dr. Claude MUVUNYI | Biomedical Services  (BIOS) | X |  |  |

After reviewing your protocol during the RNEC meeting of December 10, 2016 where quorum was met and after revisions made on the advice of the RNEC submitted on 14 February 2017 **Approval letter has been granted to the above mentioned study**

Please note that approval of the protocol and consent form is valid for **12 months.**

You are responsible for fulfilling the following requirements:

- - 1. Changes, amendments, and addenda to the protocol or consent form must be submitted to the committee for review and approval, prior to activation of the changes.
    2. Only approved consent forms are to be used in the enrollment of participants
    3. All consent forms signed by subjects should be retained on file. The RNEC may conduct audits of all study records, and consent documentation may be part of such audits.
    4. *A* continuing review application must be submitted to the RNEC in a timely fashion and before expiry of this approval.
    5. Failure to submit a continuing review application will result in termination of the study.
    6. Notify the Rwanda National Ethics committee once the study is finished.

,... •T---y'

*-*□ /<•"*'*.*\*,*f*-, *\* **1***;)***\** *R1 !J'r* '

, , j ..

*I*1.-"{:\,.*I*.*'*\:;,_

,;.j• C*•*1*..* c,e{." \,._I>

c-*_*:,*1*'.*;*\*,*..\

##### Date of Appro val: Febru a ry 20, 2017

Sincerely,

*I f3f'f · -:,.\* Expiration date: Februaryl9, 201Z,

*( ·kfi* R..NE. C l

t+,,' • .. ..

_.,;---

'\' *,r.le*

*.1...,.)../1*

'. Rwanda l ational Ethics Committee

**Dr. Jean- Baptiste MAZARATI**

**Chairperson, Rwanda National Ethics Committee.**

c.c.

##### The Hon. Minister, Ministry of Health

- The Permanent Secretary, Ministry of Health

Appro val Date:.lo l- - \ :t.

Expiration Date:f ©·f **·Q?.--. O.J**

---- - - - ·-- - - - /

REPUBLIC OF RWANDA/REPUBLIQUE DU RWANDA

M

# ..

## NATIONAL ETHICS COMMITTEE/ COMITE NATIONAL D'ETHIQUE

#### Telephone: (250) 2 55 10 78 84 Ministry of Health

**E-mail:** [**info@rnecrwanda.or1:**](mailto:info@rnecrwanda.or1) **P.O. Box. 84**

**Web site: www.rnecrwand o Kigali, Rwanda.**

**FWA Assurance No. 00001973 IRB 00001497 ofIORG0001100**

February 15, 2018 No.0075/RNEC/2018

Principal Investigator: **Sabine Musange**

Your research project: **"Annual Renewal and amendment** for **"Preterm Birth Initiative Group Care Randomized Controlled Trial"** has been evaluated by the Rwanda National Ethics committee.

|  | | Involved in the decision | | |
| --- | --- | --- | --- | --- |
|  |  | Yes | No f Reason) | |
| Name | Institute |  | Absent | Withdrawn from  the proceeding |
| Dr.Jean-Baptiste MAZARATI | Biomedical Services (BIOS) | X |  |  |
| Prof. Eugene RUTEMBESA | University of Rwanda | X |  |  |
| Dr.Laetitia NYIRAZINYOYE | University of Rwanda | X |  |  |
| Dr. Egide KAYITARE | University of Rwanda | X |  |  |
| Sr.Domitilla MUKANTABANA | Kabgayi Nursing and Midwife school | X |  |  |
| Dr. David K. TUMUSIIME | University of Rwanda | X |  |  |
| Dr. Lisine TUYISENGE | Kigali Teaching Hospital | X |  |  |
| Dr. Claude MUVUNYI | Biomedical Services (BIOS) | X |  |  |

After reviewing **amendments** to your protocol during the RNEC meeting of February 10, 2018 where quorum was met, **Continuation of approval has been granted to your study.**

Please note that approval of the protocol and consent form is valid for **12 months.**

You are responsible for fulfilling the following requirements:

- - - 1. Changes, amendments, and addenda to the protocol or consent form must be submitted to the committee for review and approval, prior to activation of the changes.
      2. Only approved consent forms are to be used in the enrollment of participants
      3. All consent forms signed by subjects should be retained on file. The RNEC may conduct audits of all study records, and consent documentation may be part of such audits.
      4. A continuing review application must be submitted to the RNEC in a timely fashion and before expiry of this approval.
      5. Failure to submit a continuing review application will result in termination of the study.
      6. Notify the Rwanda National Ethics committee once the study is finished.

Sincerely,

Date of Approval: February 10, 2018

Expiration date: February 09, 2019

**Dr. Jean- Baptiste MAZARATI**

**Chairperson, Rwanda National Ethics Committee.**

c.c.

##### Hon. Minister of Health.

- - The Permanent Secretary, Ministry of Heath
