# Supplementary material for: Assessing the impact of group antenatal care on gestational length in Rwanda: A cluster-randomized trial
Source: PLoS One. 2021 Feb 2;16(2):e0246442. doi: 10.1371/journal.pone.0246442 (PMC7853466; doi:10.1371/journal.pone.0246442)
Supplement: Attachment — Submitted filename: Response to Reviewers 2.docx [file pone.0246442.s007.docx]

PONE-D-20-10387R1
Assessing the impact of group antenatal care on gestational length in Rwanda: a cluster-randomized trial
PLOS ONE

**RESPONSE TO REVIEWERS**

**Reviewer #1: Thank you for your thoughtful comments. I've had the opportunity to review responses to my comments, and the comments of other reviewers. I'd like to congratulate you on conducting an important study with an elegant design.**

**Reviewer #2: Abstract: correct the typo in the first line of 'Findings'; I suggest it reads: "A total of 4091 women in 18 control clusters and 4752 women in 18 intervention clusters were included in the analysis..."**

Thank you for your careful attention to detail. We have corrected the typo and appreciate the suggestion.

**Background line 4: "an individually randomized" instead of "individual randomized"**

Corrected.

**Objectives: a lot of the explanations now included in the objectives, although very useful, do not belong there. For example, the following sentence should be included in the justification for the cluster-randomised design, such as in the second sentence of the 'trial design' section (words in brackets are my own suggestion): "(We opted for a clustered design because) Rwandan stakeholders preferred to offer all women at each health center the same model of care." The following sentence should also be moved to an appropriate place in the methods: "Further, at half of the health centers included in this study we implemented community-based urine pregnancy test by community health workers and basic obstetric ultrasound by nurses and midwives and conducted secondary analyses to see if these interventions affected the secondary outcomes of attendance at four antenatal care visits and initiation of antenatal care before 14 completed weeks."**

Thank you for the suggestions. In rereading we understand that some of the additions we put in in response to previous reviewer comments may have overcompensated. We further appreciate the specificity of this reviewer’s suggestions and have made the following changes:

As suggested, we moved the explanation of Rwandan stakeholders’ preference for uniform care at any health center to the trial design justification, on page 6. We have also edited this section to address only the objectives and moved the information about ultrasound and urine pregnancy section to the methods, describing in trial design the distribution of these interventions in a balanced manner on page 6-7.

**Methods: description of the sample size calculation is still inadequate. This was a cluster-randomised trial; the description should include at least two of the following: the number of clusters per arm; the number of observations per cluster; the number of individuals per arm. At the moment, only the number of individuals per arm is included, giving the reader no idea of the number of clusters per arm or observations per cluster. Please look at the reporting in other examples of studies of the same design e.g. 10.1371/journal.pmed.1001018.**

We have revised the sample size section on page 13 to reflect the underlying assumption of 36 health centers total, or 18 per study group, and an average of 202 women per cluster.

**According to your response to peer review, you are no longer doing this, therefore this should be removed from your 'statistical analysis' section: "We conducted individual level bivariate analyses stratified by study group using Chi-square and Student’s t-test statistics for categorical and continuous data, respectively, to assess study group comparability. Similar unadjusted bivariate analyses were conducted for the primary and secondary outcomes." - please go through the whole manuscript carefully to ensure that the changes you have made are reflected in the latest version and are consistent all through.**

Thank you. We apologize for the confusion. We did conduct bivariate analysis for comparability, but removed the Chi-square and t-tests, as well as all unadjusted results for outcomes, and have now thoroughly reviewed the manuscript for these inconsistencies and removed references to results that are not presented in the paper.

**Results: for the description of the sample in the first paragraph of the results, please report the mean and range or median and IQR per cluster, not mean and SD - the SD is not very meaningful in this context (fine to report SD elsewhere). Your CONSORT diagram is still incomplete - it should include the number of clusters with mean+range or median+IQR of women per cluster all through; see the CONSORT diagram in 10.1371/journal.pmed.1001018 for example.**

Thank you. We appreciate the clarifications from this reviewer and apologize for the confusion. We have replaced SDs with ranges in the first paragraph of the results section, and updated the CONSORT throughout to include cluster size and ranges at each level.

**When you report the continuous outcomes, you should use standard errors (SE) instead of standard deviations (SD); SDs are for description, SEs are for inference (and you are making inference on the outcomes).**

Thank you for the clarification. We have updated the continuous outcomes in Tables 3, S1, and S3 to provide SEs rather than SDs for continuous outcomes as well as more clearly labelling the column headers for the reader.

We have similarly cleaned up the column headers in Tables 1 and 4 for clarity.
